# Hydride-doped Ag_17_Cu_10_ nanoclusters as high-performance electrocatalysts for CO_2_ reduction

**DOI:** 10.1016/j.isci.2023.107850

**Published:** 2023-09-07

**Authors:** Xueli Sun, Peng Wang, Xiaodan Yan, Huifang Guo, Lin Wang, Qinghua Xu, Bingzheng Yan, Simin Li, Jinlu He, Guangxu Chen, Hui Shen, Nanfeng Zheng

**Affiliations:** 1 College of Energy Materials and Chemistry, Inner Mongolia University, Hohhot 010021, China; 2 School of Environment and Energy, State Key Laboratory of Luminescent Materials and Devices, Guangdong Provincial Key Laboratory of Atmospheric Environment and Pollution Control, South China University of Technology, Guangzhou 510006, China; 3 College of Chemistry and Chemical Engineering, Inner Mongolia University, Hohhot 010021, China; 4 State Key Laboratory for Physical Chemistry of Solid Surfaces, Collaborative Innovation Center of Chemistry for Energy Materials, National & Local Joint Engineering Research Center for Preparation Technology of Nanomaterials, and National Engineering Laboratory for Green Chemical Productions of Alcohols-Ethers-Esters, College of Chemistry and Chemical Engineering, Xiamen University, Xiamen 361005, China; 5 Innovation Laboratory for Sciences and Technologies of Energy Materials of Fujian Province (IKKEM), Xiamen 361102, China

**Keywords:** Catalysis, Electrochemical energy conversion, Computational chemistry, Materials science

## Abstract

The atomically precise metal electrocatalysts for driving CO_2_ reduction reactions are eagerly pursued as they are model systems to identify the active sites, understand the reaction mechanism, and further guide the exploration of efficient and practical metal nanocatalysts. Reported herein is a nanocluster-based electrocatalyst for CO_2_ reduction, which features a clear geometric and electronic structure, and more importantly excellent performance. The nanocatalysts with the molecular formula of [Ag_17_Cu_10_(dppm)_4_(PhC≡C)_20_H_4_]^3+^ have been obtained in a facile way. The unique metal framework of the cluster, with silver, copper, and hydride included, and dedicated surface structure, with strong (dppm) and labile (alkynyl) ligands coordinated, endow the cluster with excellent performance in electrochemical CO_2_ reduction reaction to CO. With the atomically precise electrocatalysts in hand, not only high reactivity and selectivity (Faradaic efficiency for CO up to 91.6%) but also long-term stability (24 h), are achieved.

## Highlights

• dppmCuBH_4_ as a reductant for the facile synthesis of metal nanoclusters
• Ag_17_Cu_10_H_4_ nanoclusters as high-performance electrocatalysts for CO_2_ reduction
• Hydrides are key in controlling electrocatalytic performance
• Metal nanoclusters as model systems for studying electrocatalytic mechanism

## Graphical abstract

**Figure:**
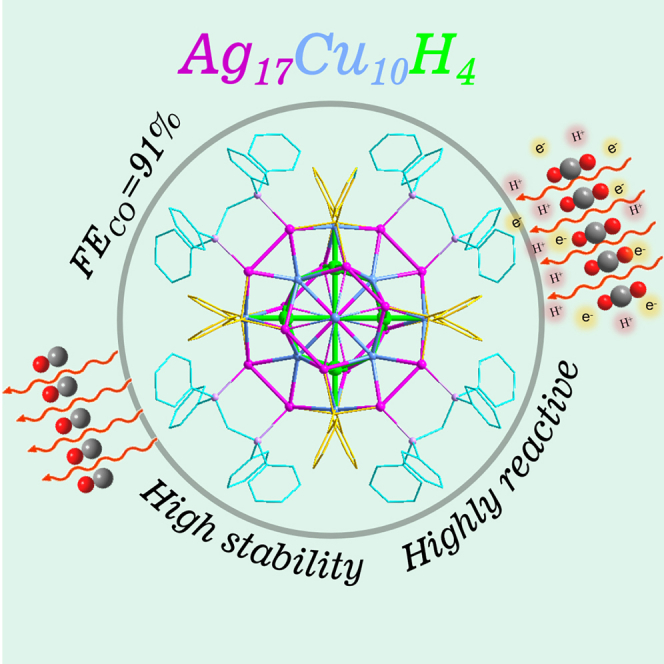

## Introduction

The reduction of carbon dioxide (CO2) via electrochemical approach has attracted much interest from fundamental research because it remains a mild and efficient way to convert CO_2_ into high value-added chemicals and balance the carbon cycle.^1^^,^^2^^,^^3^^,^^4^^,^^5^^,^^6^ Various metal nanocatalysts have been demonstrated to be high-performance catalysts in electrochemical CO2 reduction reaction (eCO_2_RR).^7^^,^^8^^,^^9^^,^^10^^,^^11^ Nevertheless, it remains a grand challenge to precisely point out the catalytic sites and deeply understand the reaction mechanism on traditional nanocatalysts, as on one hand the catalytic reaction involves multiple chemical processes and multiple products, and on the other hand the structure of most nanocatalysts is ill-defined at the atomic level.^3^^,^^4^^,^^12^^,^^13^^,^^14^^,^^15^ In this regard, atomically precise metal nanoclusters with 100% uniform size, definite composition, and molecular characteristics are evolved as model systems to investigate the structure-performance relationships of metal nanocatalysts in eCO_2_RR.^16^^,^^17^^,^^18^^,^^19^^,^^20^^,^^21^^,^^22^^,^^23^^,^^24^^,^^25^^,^^26^^,^^27^^,^^28^^,^^29^^,^^30^^,^^31^^,^^32^^,^^33^^,^^34^^,^^35^^,^^36^^,^^37^^,^^38^^,^^39^

The past few years have witnessed great progress in driving eCO_2_RR by atomically precise metal nanoclusters.^40^^,^^41^^,^^42^ The successive studies have shown that both high activity and selectivity of the reaction can be coupled in nanocluster-based catalysts and the composition, charge, shape, and surface structures of metal nanoclusters play an important role in controlling their performance.^43^^,^^44^^,^^45^^,^^46^^,^^47^^,^^48^^,^^49^^,^^50^ Close examination of the literatures reveals that metal nanoclusters used in eCO_2_RR focus on Au and only a handful of Ag and Cu ones are available.^43^^,^^44^^,^^45^^,^^46^^,^^47^^,^^48^^,^^49^^,^^51^^,^^52^^,^^53^^,^^54^^,^^55^^,^^56^^,^^57^ Among the Ag and Cu clusters, outstanding recently is the alkynyl-stabilized Ag/Cu alloy ones (Ag_15_Cu_6_ and Ag_9_Cu_6_), which have exhibited high selectivity in electroreduction of CO_2_ to CO.^58^^,^^59^ On the other hand, an increasing number of studies have highlighted the significance of hydrides in boosting the eCO_2_RR of cluster-based catalysts, with [Au_24_(NHC)_14_Cl_2_H_3_]^3+^, [Au_22_H_3_(dppe)_3_(PPh_3_)_8_]^3+^ and [Cu_32_(H)_20_{S_2_P(O^i^Pr)_2_}_12_] as the well-documented examples.^51^^,^^52^^,^^53^^,^^54^ In the continuous exploration of cluster-based catalysts, we thus wonder whether efficient eCO_2_RR catalysts can be developed by doping hydrides into the lattice of Ag/Cu alloy nanoclusters with alkynyl protection.

Herein, we report the first example of hydride-doped Ag/Cu nanoclusters as high-performance catalysts for eCO_2_RR. The cluster with the molecular formula of [Ag_17_Cu_10_(dppm)_4_(PhC≡C)_20_H_4_]^3+^ (labeled as Ag_17_Cu_10_H_4_ hereafter, dppm is bis(diphenylphosphino)methane) has been selectively obtained by dppmCuBH_4_-initiated reduction process in a simple way. The structure of the cluster as revealed by single-crystal X-ray analysis is inspiring in terms of metal framework, metal-ligand interfacial structure, and surface motifs. Notably, the cluster displays a quite high selectivity (faradaic efficiency (FE) up to 91.6%) and high stability (24 h) in eCO_2_RR to CO, outperforming most reported Ag/Cu alloy nanoclusters co-stabilized by alkynyl and phosphine ligands.

## Results and discussion

### Synthesis and atomic structure

The synthesis of Ag_17_Cu_10_H_4_ was carried out in one pot and finalized within 1 h (see method details for additional information). The simple synthetic strategy avoided any lengthy preparation steps and thus was beneficial for its later application. The synthetic parameters of the Ag_17_Cu_10_H_4_ cluster are summarized in Table 1. The key factor in the successful attainment of the title cluster is the introduction of dppmCuBH_4_ reductant in the synthesis. The dppmCuBH_4_ was prepared from ligand exchange between (PPh_3_)_2_CuBH_4_ and excess dppm. We note that this is the first time that dppmCuBH_4_ is used as reducing agent for the synthesis of atomically precise metal nanoclusters.^60^ In a typical synthesis of Ag_17_Cu_10_H_4_, PhC≡CAg suspended in mixed solvent of dichloromethane and methanol was reduced by dppmCuBH_4_. Upon the addition of reductant, the polymeric PhC≡CAg dissolved gradually, in the meanwhile the solution turned from colorless, pale yellow, pale brown to finally dark brown (Figure S1). The raw product after centrifugation was subjected to the diffusion of ether in the dark, affording black block crystals as the final product (Figure S2).

**Table 1:** Selective synthetic parameters of Ag_17_Cu_10_H_4_ cluster

Prototypes	Precursors	Solvents Reductants		Crystallization
Miscible-phase solution	PhC≡CAg	CH_2_Cl_2_ CH_3_OH	dppmCuBH_4_	Diffusion

We first performed X-ray single crystal diffraction to determine the molecular structure of the crystalline products (Figures S3 and S4). The analysis revealed that the products were crystallized in a cubic crystal system with the space group of I-$\bar{4}$ 3*m* (Table S1). In each unit cell, 6 cluster moieties are observed (Figure S5). The molecular formula of the cluster is finally determined to be [Ag_17_Cu_10_(dppm)_4_(PhC≡C)_20_H_4_]^3+^ by high-resolution electrospray ionization mass (HRESI-MS, *vide infra*), although the four hydrides are difficult to be visualized by X-ray diffraction and the three counterions have not been observed in the single crystal structure analysis. The absence of counterions in the lattice of Ag_17_Cu_10_H_4_ can be rationalized by the possibility that the anions are so disordered that cannot be distinguished or that they are not locked in the lattice.^61^

**Figure 1:**
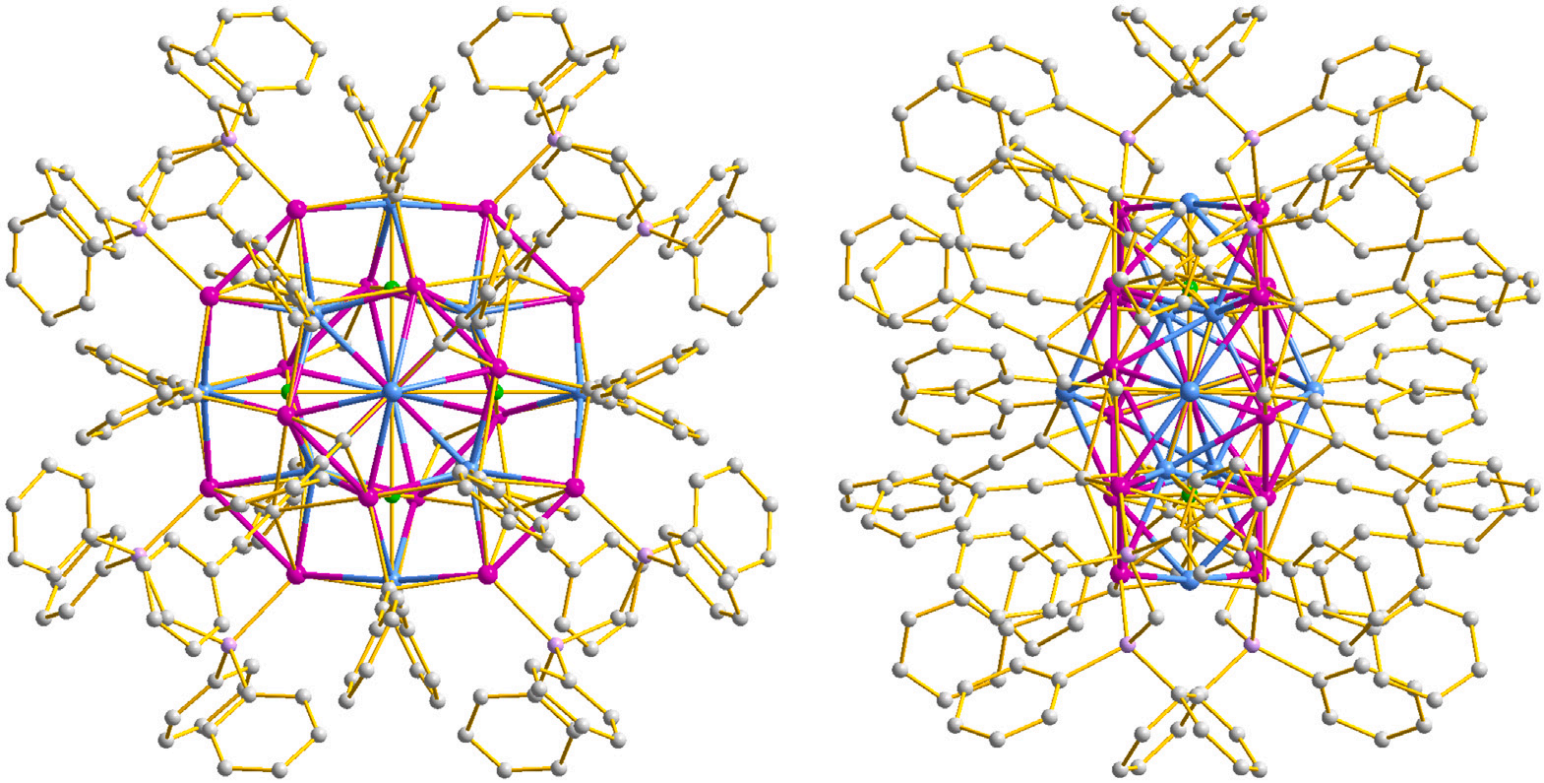
The total structure of the [Ag_17_Cu_10_(dppm)_4_(PhC≡C)_20_H_4_]^3+^ cluster along different axes Color code of atoms: pink spheres, Ag; pale blue spheres, Cu; lavender spheres, P; bright green spheres, H; gray, C.

The size of each Ag_17_Cu_10_H_4_ moiety is measured to be ∼2.0 nm (Figure S6). Shown in Figures 1 and S7 are the total structure of Ag_17_Cu_10_H_4_ along the *a, b,* and *c-*axis, respectively. The overall structure of the cluster along the *c*-axis resembles a Chinese knot made up of squares and rectangles. In the knot, the 17 Ag and 10 Cu atoms form the regular metal framework and 4 phosphine and 20 alkynyl ligands construct the shell. We note that a C_2_ rather than C_4_ symmetric axis is present in the cluster because the rectangles are in different planes (Figure S8, *vide infra*).

**Figure 2:**
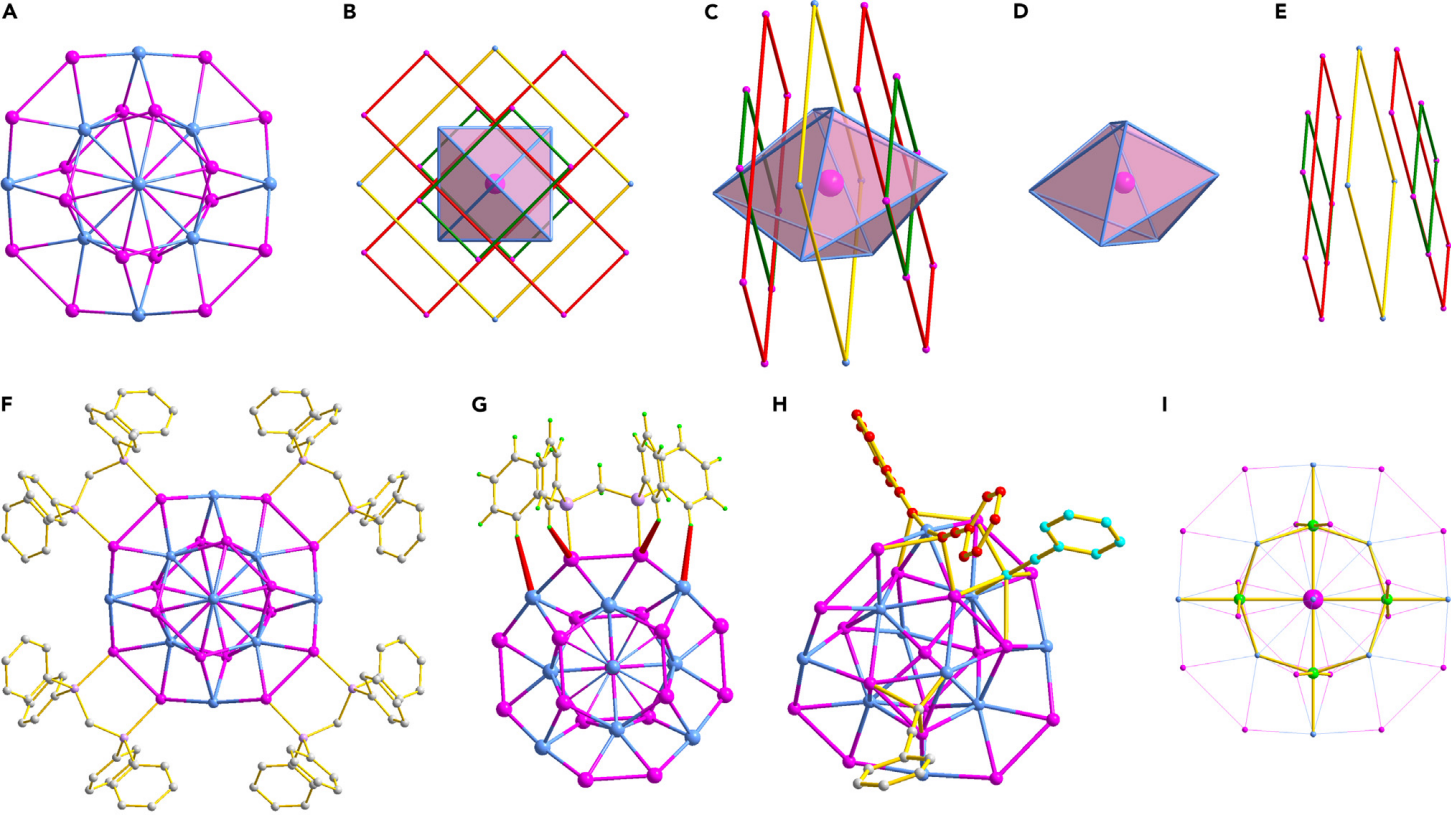
Structure anatomy of the [Ag_17_Cu_10_(dppm)_4_(PhC≡C)_20_H_4_]^3+^ cluster Color code of atoms: pink spheres, Ag; pale blue spheres, Cu; lavender spheres, P; bright green spheres, H; gray, red, and turquoise spheres, C. (A–E) The metal framework of the Ag_17_Cu_10_H_4_ cluster. (F and G) Coordination structures of dppm ligands of the Ag_17_Cu_10_H_4_ cluster. (H) Coordination modes of alkynyl ligands of the Ag_17_Cu_10_H_4_ cluster. (I) Coordination environment of hydrides of the Ag_17_Cu_10_H_4_ cluster.

The structure of the title cluster has then been anatomized carefully. Displayed in Figures 2A and S9 is the metal framework of Ag_17_Cu_10_H_4_ along different axis, whose structure can be described as the combination of several metal rings. The metal architecture of the cluster can be alternatively portrayed as the interfusion of an Ag-centered Cu_6_ polyhedron along the cavity of a Ag_4_/Ag_4_/Cu_4_/Ag_4_/Ag_4_ multi-layer partition (Figures 2B and 2C). In the Ag-centered Cu_6_ octahedron, the average bond distances of Ag-Cu and Cu-Cu give the value of 2.7405 and 3.9629 Å, respectively (Figure 2D). These values are comparable to those in other Ag/Cu alloy nanoclusters co-protected by phosphine and alkynyl ligands.^62^^,^^63^ In the Ag_4_/Ag_4_/Cu_4_/Ag_4_/Ag_4_ five-shell structure, the identical rectangles of the first and fifth Ag_4_ are arranged orthogonally, with the Ag-Ag bond lengths of the longer side 4.4160 Å and shorter 2.7920 Å, respectively (Figure 2E, marked as green). Similarly, the Ag_4_ rectangles in the second and fourth shells are orthogonal to each other as well, although their bond lengths are slightly different (longer side 9.5351 and shorter 2.9762 Å, red in Figure 2E). The remaining four Cu atoms form a perfect square with a side length of 6.473 Å, which surrounds the middle of the Cu_6_ octahedron (Figure 2E, marked as yellow).

As discussed previously, the metal core of the Ag_17_Cu_10_H_4_ cluster is stabilized by both dppm and alkynyl ligands. The four dppm ligands are connected to the metal atoms in the same coordination pattern (Figure 2F). The dppm ligands bind the Ag atoms in the outermost ring with strong interaction (average Ag-P of 2.3934 Å). Besides the Ag-P interaction, it is observed from the analysis that the dppm ligands can further solidify the metal core by multiple H-M (M = Ag or Cu) interactions (Figure 2G). As suggested by their short bond distances (2.699 Å for H-Ag and 2.865 Å for H-Cu), the hydrogen atoms of the phenyl groups in the dppm ligands bind the Ag and Cu atoms tightly (Figure S10). The 20 alkyne ligands show three coordination modes on the surface (Figure 2H). One is in μ_3_ (marked as gray), and the other in μ_5_ (marked as turquoise). The last mode refers to the “staple” motif formed by two alkynyl ligands (marked as red). The bond lengths of Ag-C and Cu-C are in the range of 2.431–2.70 Å and 1.78–2.33 Å, respectively, similar to those in [Au_13_Ag_16_(C_10_H_6_NO)_24_]^3−^, [Ag_15_(C≡C-^t^Bu)_12_]^+^ and Cu_53_(C≡CPhPh)_9_(dppp)_6_Cl_3_(NO_3_)_9_.^27^^,^^47^^,^^64^ We have also tried to propose the position of the four hydrides by using the strategy reported previously (detailed techniques see supplementary information).^65^^,^^66^^,^^67^^,^^68^ Both the experimental (crystallographic data) and theoretical (Density functional theory (DFT) calculation) results suggest the rationale of the identification (Tables S2–S5). As displayed in Figures 2I and S11, the four hydrides are symmetrically doped in the metal framework of the cluster with the same type of coordination mode. Each hydride atom is in an Ag_3_Cu_3_ octahedron (μ_6_-H), with the average bond distances of Ag-H and Cu-H of 1.9365 and 2.07 Å, respectively.

### Electronic structure and optical properties

**Figure 3:**
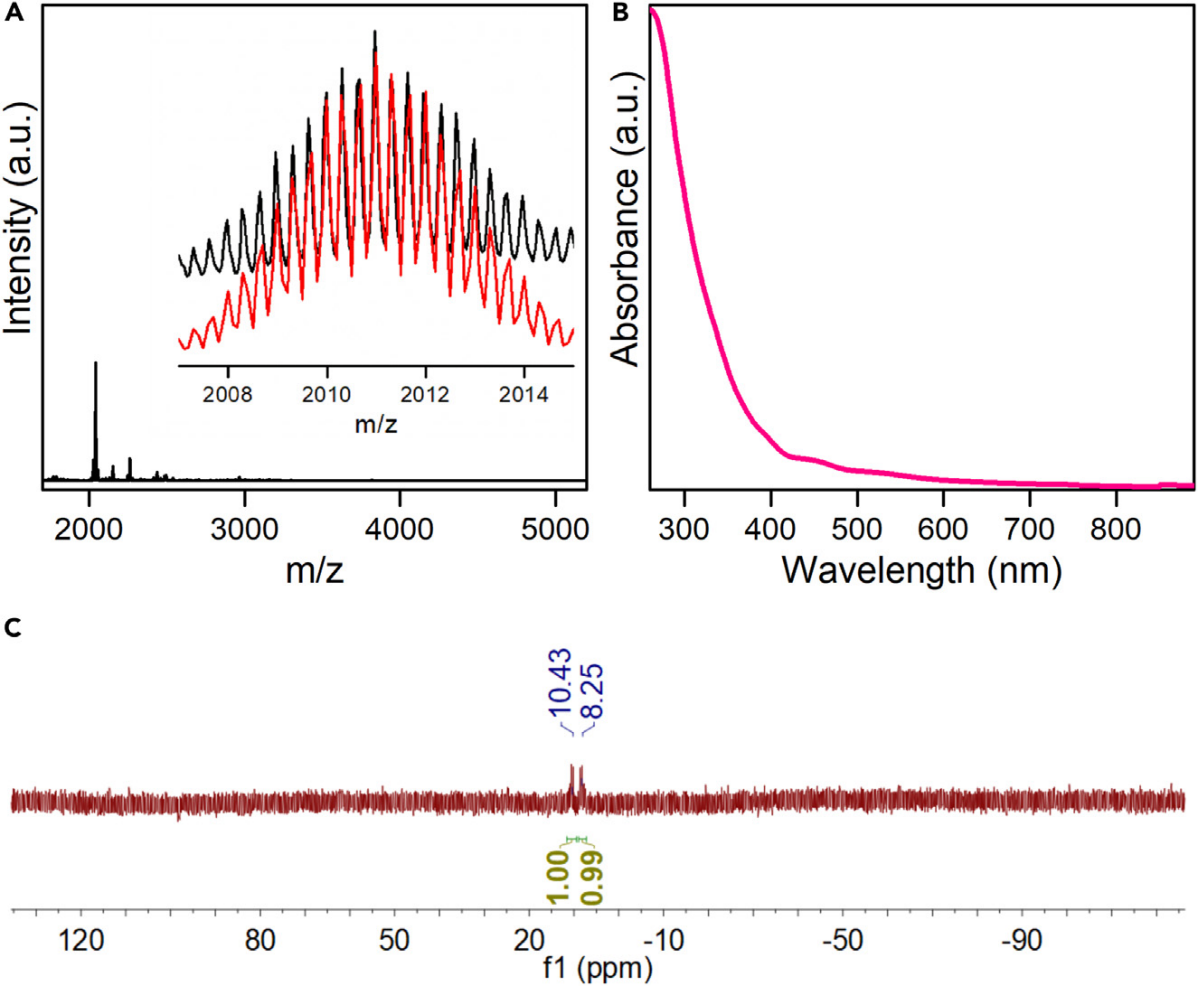
Characterization of the [Ag_17_Cu_10_(dppm)_4_(PhC≡C)_20_H_4_]^3+^ cluster (A) ESI-MS of Ag_17_Cu_10_H_4_ cluster in the positive mode. Inset: the experimental (black trace) and simulated (red trace) isotopic patterns of molecular ion peak of [Ag_17_Cu_10_(dppm)_4_(PhC≡C)_20_H_4_]^3+^. (B) UV-Vis spectra of Ag_17_Cu_10_H_4_ cluster in dichloromethane. (C) Proton-decoupled ^31^P NMR of Ag_17_Cu_10_H_4_ in CD_2_Cl_2_.

The presence of hydrides in the cluster has been unambiguously confirmed by HRESI-MS. The spectrum of the cluster in the positive measurement mode exhibits several prominent peaks at ∼2011 m/z (Figure 3A). The main peak can be assigned to [Ag_17_Cu_10_(dppm)_4_(PhC≡C)_20_H_4_]^3+^, whose experimental isotopic distribution pattern is perfectly consistent with the simulated one (Figure 3A, inset). It is noteworthy that Ag-Cu exchange is observed in the mass spectrum of the cluster, suggesting the disorder of position occupancy in the structure (Figure S12).^63^ The X-ray photoelectron spectroscopy (XPS) was then performed to confirm the valence state of Ag and Cu of Ag_17_Cu_10_H_4_ cluster. As displayed in Figure S13A, the similar XPS profiles of Ag_17_Cu_10_H_4_ with AgNO_3_ suggest that all Ag atoms in Ag_17_Cu_10_H_4_ cluster are in the +1 state. The Cu atoms in Ag_17_Cu_10_H_4_ cluster are also in oxidation state, as evidenced by its similar XPS spectra with (PPh_3_)_2_CuBH_4_ and the presence of two peaks (913.0 and 915.4 eV) in its X-ray-excited Auger electron spectroscopy (Figures S13B and S13C).^31^^,^^69^^,^^70^ Shown in Figure 3B is the experimental UV-Vis absorption spectrum of the Ag_17_Cu_10_H_4_ cluster in CH_2_Cl_2_ at room temperature. It exhibits three absorption bands at 390, 446 and 524 nm, respectively. The solution behavior of the Ag_17_Cu_10_H_4_ cluster has also been investigated by nuclear magnetic resonance (NMR, Figure S14). Proton-decoupled ^31^P NMR of Ag_17_Cu_10_H_4_ cluster in CD_2_Cl_2_ exhibits two groups of heptets centered at 9.387 ppm, suggesting that the cluster retains its molecular moieties in the solution from (Figure 3C).^71^

**Figure 4:**
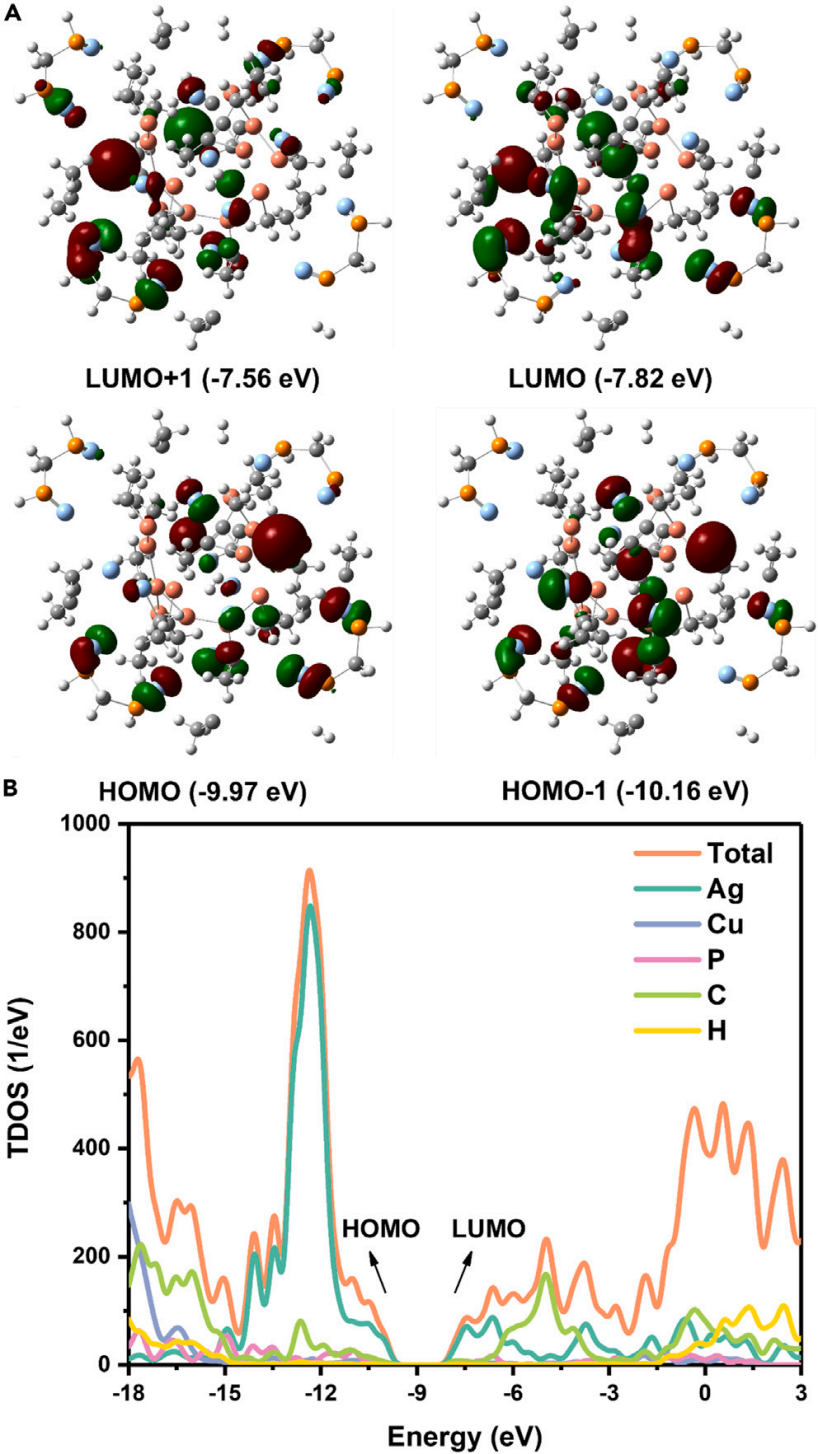
Electronic structure of [Ag_17_Cu_10_(dppm)_4_(PhC≡C)_20_H_4_]^3+^ studied by DFT calculation (A) Frontier orbitals of Ag_17_Cu_10_H_4_. (B) PDOS of Ag_17_Cu_10_H_4_.

DFT computations were then performed to gain deep insight into the electronic properties of the Ag_17_Cu_10_H_4_ cluster and corroborate the rationality of hydride locations proposed from X-ray crystallographic analysis. The electronic structure calculations of the Ag_17_Cu_10_H_4_ cluster were done using the semi-empirical method PM6 of the Gaussian 09 package (see the supplementary information text for details). The geometry optimization was started from the experimentally measured structure, which did not change the atomic arrangement of the cluster. The atomic coordinates and averaged bond lengths of optimized structures are summarized in Tables S2–S4. The obtained data show that the averaged bond lengths are consistent well with the experimental values (Table S5). Figure 4A and Table S6 are the frontier orbital charge densities of the Ag_17_Cu_10_H_4_ cluster. It can be seen that the charge densities are primarily localized near Cu atoms. This indicates that Cu atoms are the catalytic active sites in the Ag_17_Cu_10_H_4_ cluster. We have also calculated the Bader charge to analyze the charge transfer between elements of Ag_17_Cu_10_H_4_ cluster (Table S7).^72^^,^^73^ In general, each of the Ag, Cu, and P atoms in Ag_17_Cu_10_H_4_ cluster lose electrons and carry positive charges, with an average amount of 0.309 electrons for Ag, 0.438 for Cu, and 1.286 for P, respectively. For C and H, the situation is different, as they are unevenly charged. For example, the H atoms inside the cluster and around the P atoms hold more negative charge, while the charges carried by other H atoms are positive. This indicates that the H inside the cluster is electro-withdrawing (namely, hydride). From the analysis of the Bader charge, it is reasonable to assume that the electrons of Ag and Cu are transferred to nearby C and H. Cu transfers more electrons, so it is more active than Ag. Normally, the positive-charged site can act as Lewis acid, so the Cu site is the better site for the activation of Lewis bases (such as CO_2_). Figure 4B are the projected density of states (PDOS) of the Ag_17_Cu_10_H_4_ cluster. Seen from the PDOS, the energy gap between HOMO and LUMO is 2.15 eV, and the HOMO and LUMO orbitals of Ag_17_Cu_10_H_4_ cluster are mainly provided by the Cu and Ag elements, which agrees well with the orbital charge densities in Figure 4A.

### Electrocatalytic CO_2_ reduction

Excellent catalytic performance (high activity, selectivity, and stability) is highly desired for cluster-based catalysts when driving eCO_2_RR. The following features of the Ag_17_Cu_10_H_4_ cluster enable it a promising candidate catalyst for eCO_2_RR: (1) the enhanced stability brought by both large electronic energy gap and multiple coordination interactions between metal framework and dppm ligands; (2) the ready exposure of metal active sites from the removal of labile alkynyl ligands under the electrocatalytic conditions^47^^,^^59^^,^^74^; (3) the unique local surface structure of the Ag_17_Cu_10_H_4_ cluster and the inclusion of hydride species in its framework that may guide the catalytic processes taken place in a specific way and enhance the selectivity. We thus in the following section set out to explore the catalytic performance of the Ag_17_Cu_10_H_4_ cluster in eCO_2_RR.

To enhance the dispersion of cluster catalysts and overcome the conductivity problems, the Ag_17_Cu_10_H_4_ cluster was first deposited on carbon black (XC-72R) with a loading of 1 wt % to form the catalysts of Ag_17_Cu_10_H_4_/XC-72R (Figure S15). The eCO_2_RR measurement was conducted in a flow cell equipped with a two-electrode system using iridium oxide sprayed on titanium mesh as a counter electrode (Figure S16). In the cell configuration, the cathode and anode were separated by a Sustainion membrane. CO_2_ was passed through the cathode chamber at a flow rate of 40 sccm, while 1 M KOH electrolyte was circulated through the anode chamber and cathode chamber by a peristaltic pump. The produced gas and liquid products were analyzed by gas chromatography and ^1^H NMR (Figure S17).

**Figure 5:**
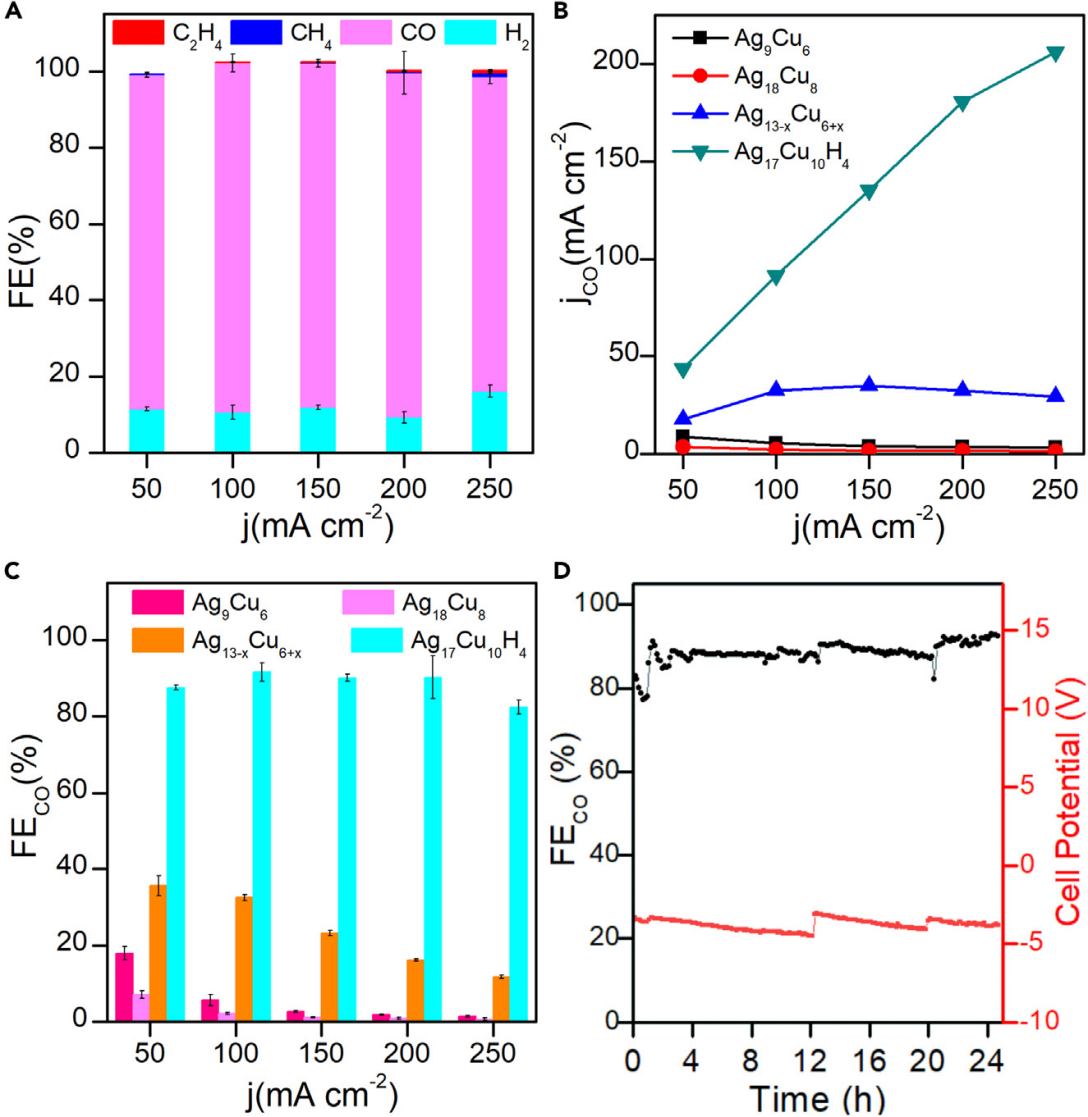
Electrocatalytic CO_2_ reduction studies of [Ag_17_Cu_10_(dppm)_4_(PhC≡C)_20_H_4_]^3+^ cluster (A) FE values of all products on Ag_17_Cu_10_H_4_ cluster under different applied current densities. (B) Partial current density to CO on Ag_9_Cu_6_, Ag_18_Cu_8_ cluster, Ag_6-x_Cu_6+x_, and Ag_17_Cu_10_H_4_ clusters under different applied current densities. (C) FE values of CO on Ag_9_Cu_6_, Ag_18_Cu_8_, Ag_6-x_Cu_6+x,_ and Ag_17_Cu_10_H_4_ clusters under different applied current densities. (D) eCO_2_RR stability measurement of CO during 24 h of electrolysis with an applied current density of 100 mA cm^−2^.

As envisioned, the Ag_17_Cu_10_H_4_ cluster exhibits excellent performance in eCO_2_RR to CO. The product distribution of eCO_2_RR on Ag_17_Cu_10_H_4_ in the current density range (50–250 mA cm^−2^) is shown in Figure 5A. Impressively, CO FE (FE_CO_) in all tested current densities is higher than 80%, with the highest FE_CO_ up to 91.6% at 100 mA cm^−2^. In comparison to other Ag/Cu alloy nanoclusters co-stabilized by alkynyl and phosphine ligands but without hydrides, the Ag_17_Cu_10_H_4_ cluster exhibits superior performance when used as a catalyst for eCO_2_RR to CO. For example, the mass activity of Ag_17_Cu_10_H_4_ cluster is calculated to be 41 A/mg, much higher than that of [Ag_15_Cu_6_(C≡CR)_18_(DPPE)_2_]^−^ reported by Hyeon et al. (0.5 A/mg). Moreover, displayed in Figures 5B and 5C is the comparison of the partial current density to CO (j_CO_) and FE values of CO on Ag_9_Cu_6_, Ag_18_Cu_8_, Ag_13-x_Cu_6+x_, and Ag_17_Cu_10_H_4_ clusters, respectively.^58^^,^^62^^,^^63^ Evidently, both j_CO_ and FE_CO_ show that the Ag_17_Cu_10_H_4_ cluster features much better selectivity than other clusters. For example, Ag_17_Cu_10_H_4_ shows the highest j_CO_ among the four clusters at all investigated current densities. The j_CO_ of the Ag_17_Cu_10_H_4_ cluster at 250 mA cm^−2^ can be high up to 206.25 mA cm^−2^ and is nearly 7, 60, and 140 times than that of Ag_13-x_Cu_6+x,_ Ag_9_Cu_6_, and Ag_18_Cu_8_ counterparts, respectively. The Ag_17_Cu_10_H_4_ cluster correspondingly exhibits much higher FE_CO_ than the comparative clusters (Figure 5C), indicating that H_2_ evolution can be significantly suppressed on the Ag_17_Cu_10_H_4_ cluster. The excellent catalytic performance of Ag_17_Cu_10_H_4_ cluster may be attributed to multiple factors, including but not limited to, the presence of both Ag and Cu active sites, the inclusion of hydride species in near the surface metal atoms, and the unique geometric and electronic structures. The stability of the Ag_17_Cu_10_H_4_ cluster in eCO_2_RR has also been evaluated by monitoring the products at a constant current density of 100 mA/cm^2^ over 24 h. As portrayed in Figure 5D, the FE_CO_ remains unchanged (∼90%) during the test. In the meanwhile, stable full cell potentials have been observed over the period. The Chronopotentiometry test data of Ag_17_Cu_10_H_4_ cluster at various current densities are shown in Figure S18, which show that the Ag_17_Cu_10_H_4_ cluster has a stable full cell voltage over the entire test current density range. The unchanged UV-Vis profiles of the Ag_17_Cu_10_H_4_ cluster after catalysis is another strong evidence for its high robustness in the eCO_2_RR (Figure S19).

**Figure 6:**
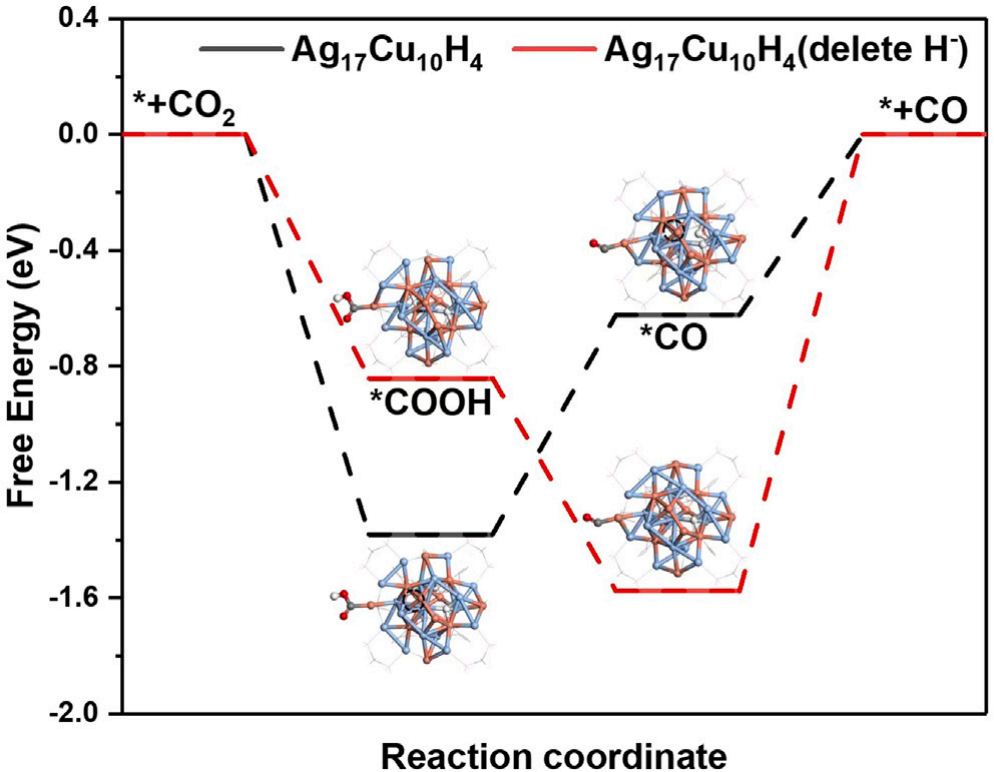
Free energy profiles of electrochemical CO_2_ reduction reaction on the Ag_17_Cu_10_H_4_ cluster for the production CO

To further investigate the reactive mechanism and rationalize the high selectivity of CO_2_ reduction reaction over the Ag_17_Cu_10_H_4_ cluster, we have performed the DFT calculations using the optimized structures (Figure 6). Here, we construct two types of clusters, including pristine Ag_17_Cu_10_H_4_ cluster (black trace) and Ag_17_Cu_10_H_4_ cluster with the absence of hydrides, called Ag_17_Cu_10_ delete H^−^ (red trace). The light blue, orange, gray, red, and white balls represent Ag, Cu, C, O, and N atoms, respectively. During the simulations, we considered different adsorption sites (Cu, Ag, Cu-Ag sites) and found that the intermediates (∗COOH, ∗CO) can be stably present and adsorbed on the clusters only when they are on the Cu site (Figures S20 and S21). In the pristine cluster, the determining step of CO_2_RR is the generation of ∗CO, and the energy barrier is 0.76 eV. In contrast, the rate-determining step of CO_2_RR for the Ag_17_Cu_10_ delete H^−^ system is the desorption of CO, and the energy barrier of determining step increase to 1.58 eV, which indicates the presence of hydrides is beneficial for the formation of CO products. The results, together with previous reports by others, demonstrate the key role of hydrides in controlling the reaction pathway and product selectivity.^52^^,^^53^^,^^54^^,^^75^ It also inspires the exploration of hydride-doped metal nanoclusters as high-performance electrocatalysts for CO_2_ reduction in future studies.

### Conclusion

In conclusion, a simple synthetic approach of using dppmCuBH_4_ as a reductant has been developed for the access to hydride-doped Ag/Cu alloy nanoclusters co-protected by phosphine and alkynyl ligands. The co-presence of silver, copper, and hydrides in the framework, along with the stabilization effect from the dppm and phenylacetylene ligands endows the [Ag_17_Cu_10_(dppm)_4_(PhC≡C)_20_H_4_]^3+^ cluster with unique geometric and electronic structures. The multiple binding interactions from dppm make the cluster robust, while the surface alkynyl ligands are flexible enough to expose Cu active sites. The included hydride species are proposed to help transfer protons and electrons. As a result, the cluster catalyst exhibits high reactivity and selectivity (FE_CO_ up to 91.6%) and long-term stability (24 h) in electrochemical CO_2_ reduction reaction to CO. The current work not only provides atomic insights into the composition/structure-performance relationships of metal nanoclusters in electrochemical CO_2_ reduction but also inspires the exploration of more underlying metal nanocatalysts with excellent electrocatalytic properties by using the strategies learned from atomically precise nanochemistry.

### Limitations of the study

The valence state of the catalyst after the reaction cannot be discussed in detail in the work.

Low XPS signal from low loading prohibits identification of valence state of the catalyst after the reaction. Furthermore, different attempts to measure active electrode surface area of catalysts prove unsuccessful.

## STAR★Methods

### Key resources table

**Table:** 

REAGENT or RESOURCE	SOURCE	IDENTIFIER
**Chemicals, peptides, and recombinant proteins**		

bis(diphenylphosphino)methane	J&K Scientific Ltd	CAS: 2071-20-7
triphenylphosphine	J&K Scientific Ltd	CAS: 603-35-0
sodium hexafluoroantimonate	Adamas	CAS: 16925-25-0
sodium borohydride	Adamas	CAS: 16940-66-2
copper(I) chloride	Innochem	CAS: 7758-89-6
silver nitrate	Innochem	CAS: 7761-88-8
phenylacetylene	Alfa Aesar	CAS: 536-74-3
triethylamine	Sinopharm Chemical Reagent Co. Ltd.	CAS: 2011-11-2
dichloromethane	Sinopharm Chemical Reagent Co. Ltd.	CAS: 75-09-2
methanol	Sinopharm Chemical Reagent Co. Ltd.	CAS: 67-56-1
ether	Sinopharm Chemical Reagent Co. Ltd.	CAS: 60-29-7

### Resource availability

#### Lead contact

Further information and requests for resources should be directed to and will be fulfilled by the lead contact, Hui Shen (shen@imu.edu.cn).

#### Materials availability

Water used in all experiments was ultrapure. All other reagents were used as received without further purification. (PPh_3_)_2_CuBH_4_, dppmCuBH_4_ and PAAg were prepared according to literature methods.^60^^,^^76^

### Method details

#### Synthesis

Synthesis of [Ag_17_Cu_10_(dppm)_4_(PhC≡C)_20_H_4_]^3+^

In a typical synthesis, PAAg (5 mg, 0.026 mmol) was suspended in the solvents of dichloromethane (1.5 mL) and methanol (0.5 mL). The mixture was treated under ultrasonication for 5 min. Then the freshly prepared reducing agent of dppmCuBH_4_ (6 mg) in dichloromethane was added once. The polymeric PAAg was dissolved gradually, with the solution color turning from colorless, pale yellow to finally dark black. Then the reaction was aged at room temperature for 12 h. After that, the solution was dried up on a rotary evaporator to afford black powder. The obtained powder was then washed with hexane and ether. After drying, dichloromethane was used to dissolve the solid, and the resultant brown solution was filtered. The raw solution was subjected to the diffusion of ether and black block crystals were obtained after ∼2 weeks.

#### Characterizations

UV-Vis data of [Ag_17_Cu_10_(dppm)_4_(PhC≡C)_20_H_4_]^3+^ was recorded on a JascoV-780 Spectrophotometer from 200 to 900 nm at room temperature. The cluster was dissolved in dichloromethane and the signal from the blank solvent was subtracted from the measurement. The data interval was 1 nm and the scan speed was 1000 nm/min.

Time-of-flight electrospray ionization mass data (TOF-ESI-MS) of [Ag_17_Cu_10_(dppm)_4_(PhC≡C)_20_H_4_]^3+^ was recorded using an Agilent 6224 time-of-flight mass spectrometer in the positive mode. The cluster was dissolved in dichloromethane and filtered before the measurement. Then the diluted solution was directly infused into the spectrometer by a syringe pump at a flow rate of 1.2 mL/h. The typical parameters for the data collection were as follows: capillary voltage: 4.0 kV; drying gas temp: 150°C; drying gas flow: 4 L/min; and nebulizer pressure: 20 psi.

^1^H and ^31^P NMR spectra were collected at room temperature on a Bruker AV-600, spectrometer with TMS and solvent residual signal as an internal reference. The NMR data were processed on MestReNova software.

The X-ray photoelectron spectroscopic (XPS) data of [Ag_17_Cu_10_(dppm)_4_(PhC≡C)_20_H_4_]^3+^, AgNO_3_ and (PPh_3_)_2_CuBH_4_ were collected on ESCALABXI+ System (Thermo Fisher Scientific, U.K). The C 1s peak of adventitious carbon was used for position correction (284.8 eV) in all cases.

The crystal morphologies of the copper cluster samples were characterized on a transmission electron microscope (Talos F200C G2, 200 KV). The TEM specimens were prepared by dropping an ether suspension of copper nanoparticle sample onto a copper grid. The particle sizes of the copper clusters were directly measured from the TEM images of at least 150 individual particles.

The diffraction data of the single crystals of [Ag_17_Cu_10_(dppm)_4_(PhC≡C)_20_H_4_]^3+^ cluster was recorded on a Rigaku Oxford Diffraction system X-ray single-crystal diffractometer using Cu Kα (λ = 1.54184 Å) at 100 K. The data were processed using CrysAlis^Pro^. The structure was solved and refined using Full-matrix least-squares based on F^2^ using ShelXT,^77^ ShelXL^78^ in Olex2,.^79^ The hydrogen atoms of organic ligands were generated geometrically. Note: the counterions of the cluster could not be observed by X-ray crystallography, which is maybe due to the missing of the anions in the lattice.^61^ The formula of [Ag_17_Cu_10_(dppm)_4_(PhC≡C)_20_H_4_]^3+^ was confirmed by mass spectrometry. The thermal ellipsoids of the ORTEP diagram were done at 50% probability. Detailed crystal data and structure refinements for the compound are given in Table S1. CCDC 2256124 contains the supplementary crystallographic data for this paper. Further details can be obtained from the CIF files deposited at the Cambridge Crystallographic Data Center and can be obtained free of charge on request via http://www.ccdc.cam.ac.uk/data_request/cif.

#### DFT calculations

The geometry optimization of the isolated Ag_17_Cu_10_H_4_ cluster is calculated using the semi-empirical method PM6 of the Gaussian 09 package. Based on the optimized Ag_17_Cu_10_H_4_ cluster, the single-point energy of the structure is calculated using the PBE method, and the projected density of states (PDOS) is obtained by the Multiwfn package.^80^ The reaction scheme for CO_2_RR is performed using the Vienna Ab initio Simulation Package (VASP).^81^ The exchange−correlation interactions are described via the Perdew-Burke-Ernzerhof (PBE) functional.^82^ The interactions between the ionic cores and the valence electrons are treated with the projector augmented wave (PAW) method.^83^ The energy cutoff is set to 500 eV. To save computational cost, we simplified the benzene ring (Ph) of the Ag_17_Cu_10_ cluster to H. as done by others.^48^^,^^61^

The Gibbs free energy G is computed using the following equation:

$$G=E+E_{ZPE}-TS-eU$$

Here, E, $E_{ZPE}$, and S represent the single point energy, zero-point energy and entropy, respectively. U denotes the potential versus standard hydrogen electrode. T is set to 298.15 K.

#### Working electrode preparation

The CH_2_Cl_2_ solutions of nanoclusters were added to pre-dispersed XC-72R in large quantities of ethanol. After stirring overnight, the solvent was removed collection by centrifugation. The XC-72R-supported clusters (1 wt %) were used to prepare catalyst inks. For the flow cell test, 2 mg of XC-72R-supported clusters, 20 μL of Nafion, and 1 mL of ethanol were mixed and sonicated for 5 min. The prepared catalyst ink was sprayed on the carbon paper, and the loading amount was controlled to be 0.5 mg cm^−2^. The geometric area of the working electrode was masked to be 1 cm^2^.

#### Electrochemical CO_2_ reduction reaction (eCO_2_RR) measurement

The eCO_2_RR performance in the flow cell was a two-electrode system using IrO_2_ sprayed on Ti mesh as a counter electrode. The cathode and anode were separated by an anion exchange membrane (Fumasep, FAA-3-PK-130). CO_2_ was passed through the cathode chamber at a flow rate of 40 sccm, while 1 M KOH electrolyte was circulated through the anode chamber and cathode chamber by a peristaltic pump. We used Chronopotentiometry (CP) method to test electrochemical CO_2_ reduction performance. The produced gas products from the cathode chamber were injected and analyzed by the GC online. The liquid products were analyzed by ^1^H NMR by using d_6_-DMSO as the internal standard. Using a calibration curve of each product, the total FE was confirmed to be close to 100%.

The gaseous products of eCO_2_RR (H_2_, CO, CH_4_, C_2_H_4_, and C_2_H_6_) were analyzed by online-connected gas chromatography (GC-2014C, SHIMADZU) equipped with a six-port sampling valve and a TDX-01 packed column. Ar (99.999%) was used as a carrier gas. H_2_ gas was quantified with a thermal conductivity detector (TCD) and the other gases were quantified with a flame ionization detector (FID). The sampling gas was passed through a mechanized before FID detector when a low concentration of CO gas was quantified. The faradaic efficiency (FE) of a given product was calculated by the following equation.

FE=nFvrP/iRT

where n is the number of electrons transferred, F is the Faraday constant, v is the CO_2_ flow rate, r is the concentration of the gas product in parts-per-million (ppm), P is the pressure, i is the total current, R is the ideal gas constant and T is temperature.

For example, when using 100 mA constant current electrolysis for 30 min, three points were collected chromatographically. The CO concentrations were 16901.48 ppm, 17787.22 ppm, and 177543.96 ppm, respectively. And the H_2_ concentrations were 2415.21 ppm, 1761.77 ppm, 1881.76 ppm, respectively. After substituting the flow rate, current, and concentration into the above formula, the FE of H_2_ (12.71, 9.27, 9.9) and CO (88.92, 93.58, 92.3) were obtained. Finally, the calculated FE efficiency of H_2_ and CO is averaged to be 10.6 and 91.6, respectively. Note that the gas flow velocity unit SCCM should be converted to mL/s.

## Data Availability

The accession number for the [Ag_17_Cu_10_(dppm)_4_(PhC≡C)_20_H_4_]^3+^ crystal structure cif. file reported in this paper is CCDC: 2256124. The data supporting the findings of this study are available in the article and Supplemental Items or from the corresponding authors upon reasonable request.
