# Supplementary material for: Hydride-doped Ag17Cu10 nanoclusters as high-performance electrocatalysts for CO2 reduction
Source: iScience. 2023 Sep 7;26(10):107850. doi: 10.1016/j.isci.2023.107850 (PMC10518712; doi:10.1016/j.isci.2023.107850)
Supplement: Document S1. Figures S1–S21 and Tables S1–S7 [file mmc1.pdf]

iScience, Volume *26*

Supplemental information

Hydride-doped $Ag_{17}Cu_{10}$ nanoclusters as high-performance electrocatalysts for CO_2 reduction

Xueli Sun, Peng Wang, Xiaodan Yan, Huifang Guo, Lin Wang, Qinghua Xu, Bingzheng Yan, Simin Li, Jinlu He, Guangxu Chen, Hui Shen, and Nanfeng Zheng

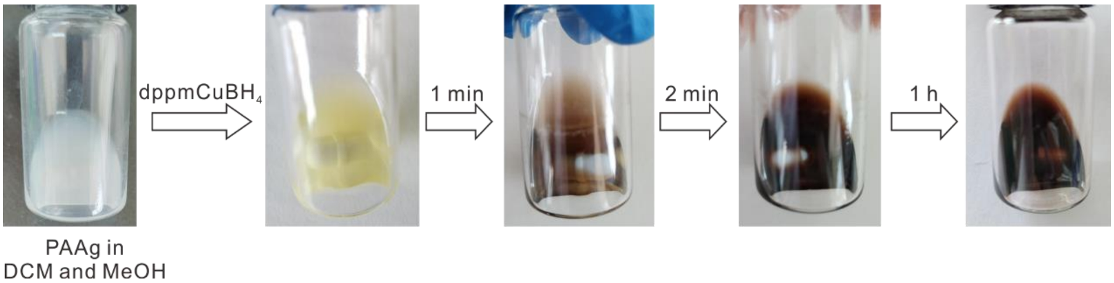

Figure S1. Digital photographs showing the synthetic process of the $Ag_{17}Cu_{10}H_4$ cluster, related to the STAR Methods.

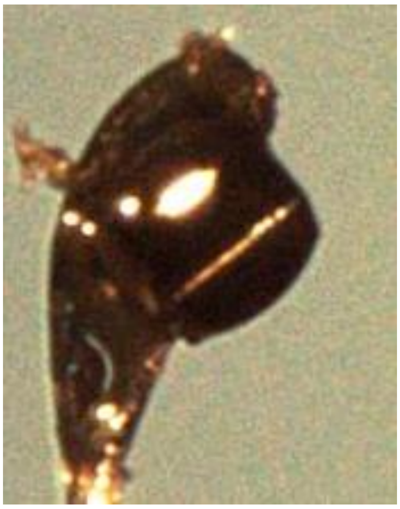

Figure S2. Digital photographs of single crystals of the $Ag_{17}Cu_{10}H_4$ cluster, related to the Figure 1.

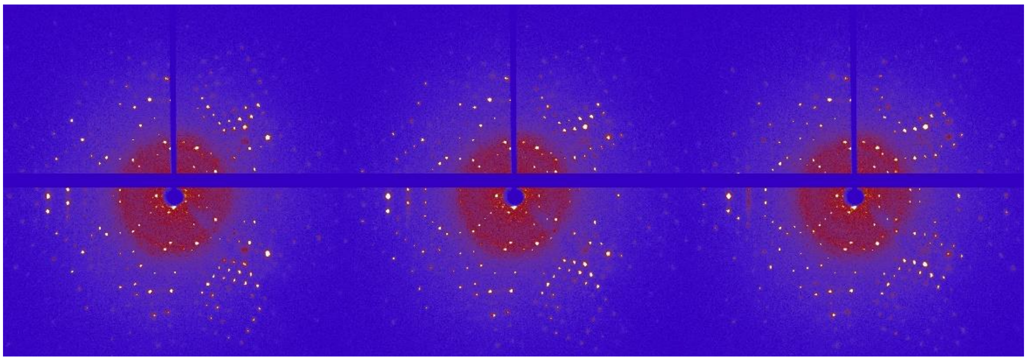

Figure S3. Representative single crystal diffraction patterns of $Ag_{17}Cu_{10}H_4$ cluster, related to the Figure 1.

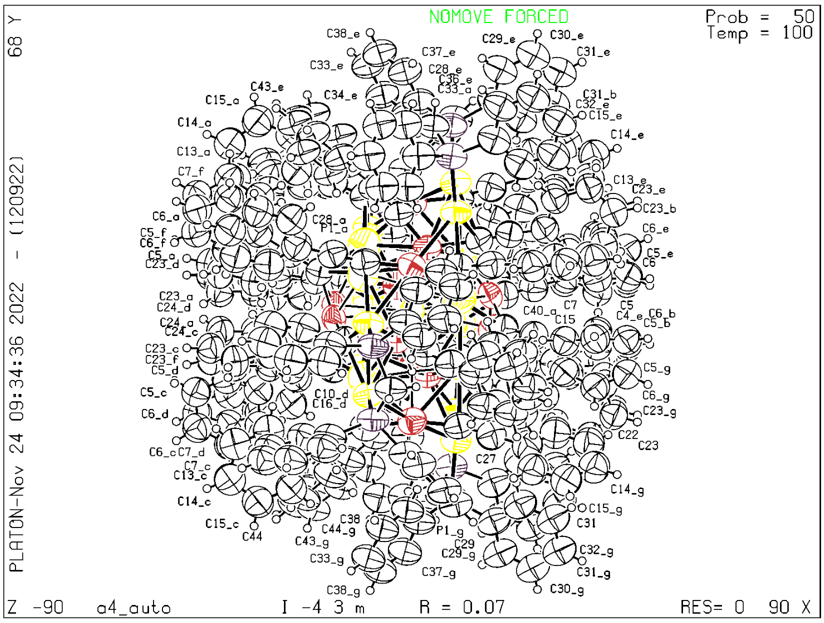

Figure S4. The thermal ellipsoids of the ORTEP diagram of $Ag_{17}Cu_{10}H_4$, related to the Figure 1.

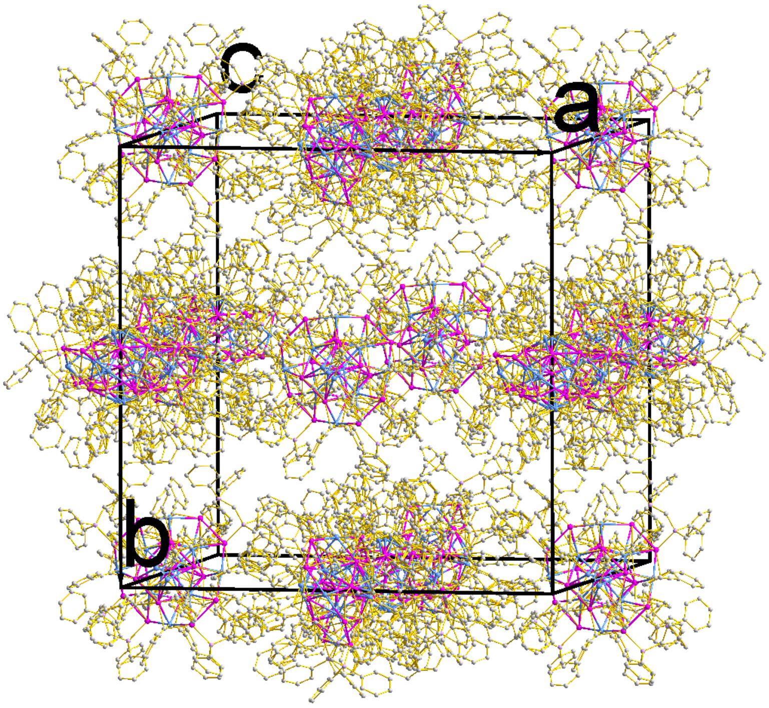

Figure S5. The packing structure of the $Ag_{17}Cu_{10}H_4$ cluster in the unit cell, related to the Figure 1. Color codes for atoms: red spheres, Ag; blue spheres, Cu; pink spheres, P; grey spheres, C. All hydrogen atoms are omitted for clarity.

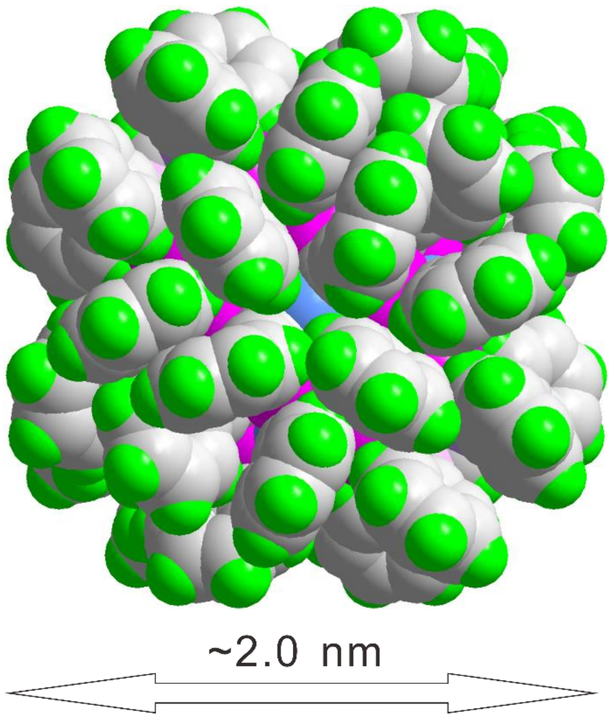

Figure S6. Total structure of $Ag_{17}Cu_{10}H_4$, related to the Figure 1. Color legend: orange spheres, Au; blue spheres, Cu; pink spheres, P; turquoise, Cl; gray spheres, C. All hydrogen atoms are omitted for clarity.

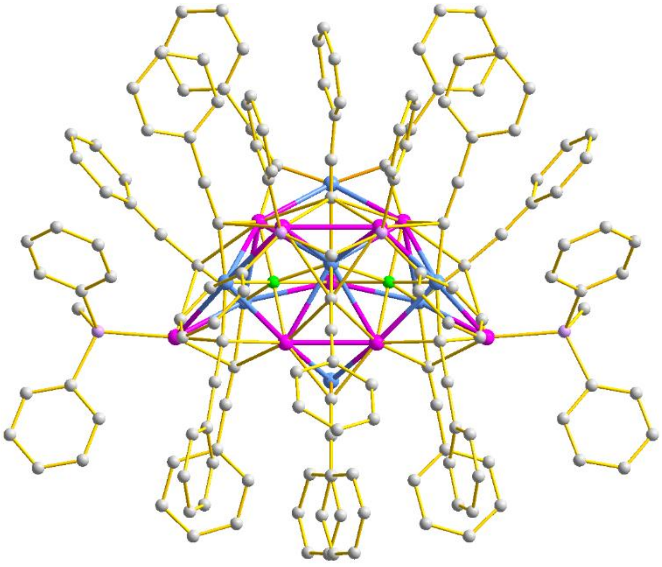

Figure S7. The total structure of $Ag_{17}Cu_{10}H_4$ cluster in the top and side views, related to the Figure 1. Color codes for atoms: red spheres, Ag; blue spheres, Cu; pink spheres, P; grey spheres, C. All hydrogen atoms are omitted for clarity.

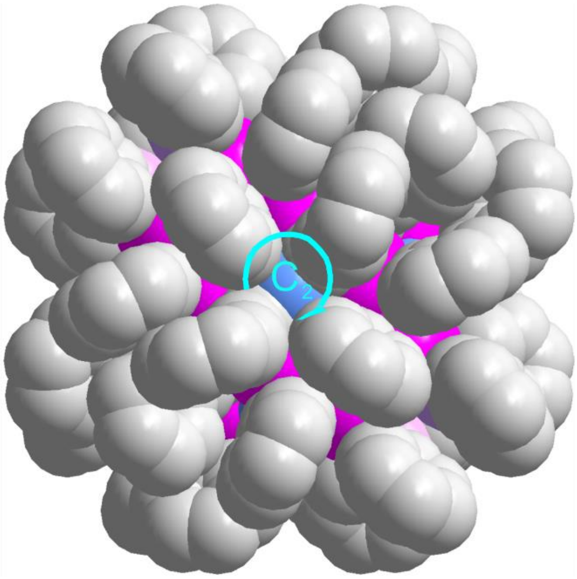

Figure S8. A C_2 axis is present in the structure of $Ag_{17}Cu_{10}H_4$, related to the Figure 1. Color legend: orange spheres, Au; blue spheres, Cu; pink spheres, P; turquoise, Cl; gray spheres, C. All hydrogen atoms are omitted for clarity.

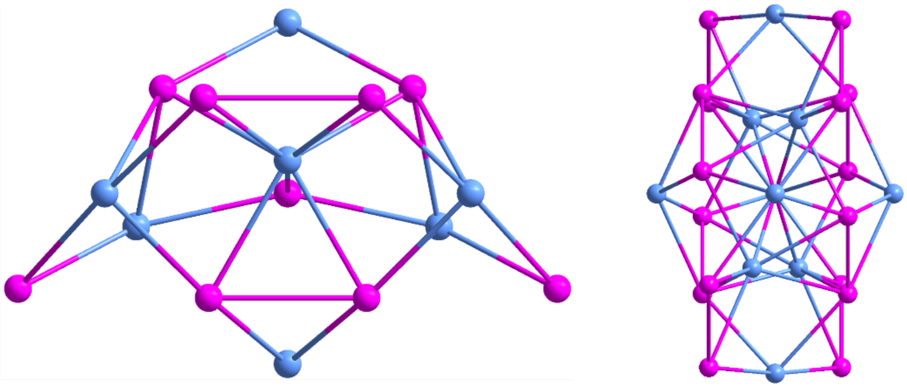

Figure S9. The total structure of $Ag_{17}Cu_{10}H_4$ cluster in the top and side views, related to the Figure 2. Color codes for atoms: red spheres, Ag; blue spheres, Cu; pink spheres, P; grey spheres, C. All hydrogen atoms are omitted for clarity.

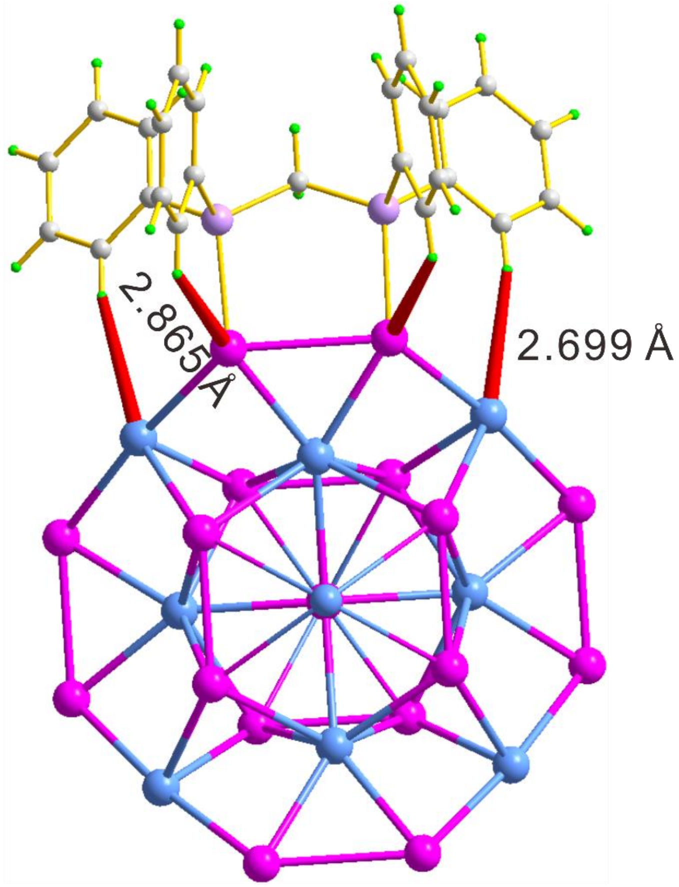

Figure S10. The total structure of $Ag_{17}Cu_{10}H_4$ cluster in the top and side views, related to the Figure 2. Color codes for atoms: red spheres, Ag; blue spheres, Cu; pink spheres, P; grey spheres, C. All hydrogen atoms are omitted for clarity.

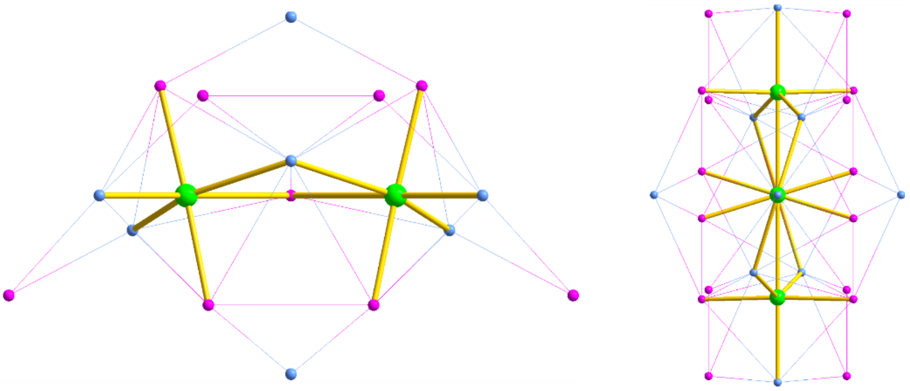

Figure S11. The total structure of $Ag_{17}Cu_{10}H_4$ cluster in the top and side views, related to the Figure 2. Color codes for atoms: red spheres, Ag; blue spheres, Cu; pink spheres, P; grey spheres, C. All hydrogen atoms are omitted for clarity.

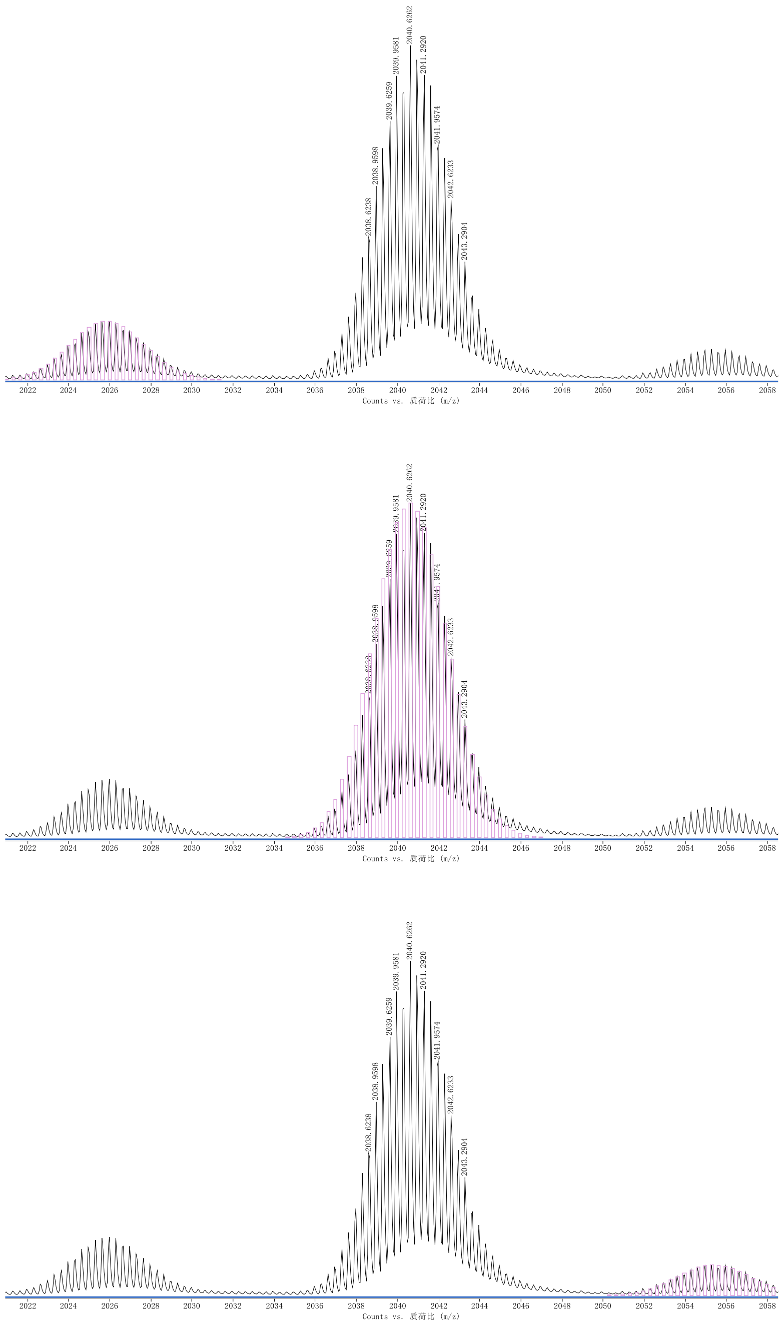

Figure S12. The presence of Ag-Cu exchange in ESI-MS spectra of $Ag_{17}Cu_{10}H_4$, related to the Figure 3. From top to bottom are the experimental and simulated isotopic patterns of the molecular ion peak $[Ag_{16+x}Cu_{11-x}(dppm)_4(PA)_{20}H_4]^{3+}$ (x=0-2), respectively.

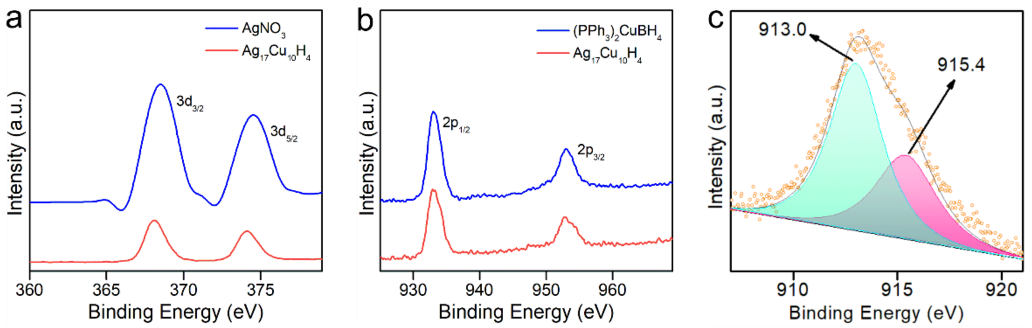

Figure S13. XPS data analysis of $Ag_{17}Cu_{10}H_4$ cluster, related to the Figure 3. Comparison of high-resolution XPS spectra of $Ag_{17}Cu_{10}H_4$ cluster and $AgNO_3$ (a), $Ag_{17}Cu_{10}H_4$ cluster and $(PPh_3)_2CuBH_4$ (b). (c) Cu LMM X-ray-excited Auger electron spectroscopy of $Ag_{17}Cu_{10}H_4$ cluster.

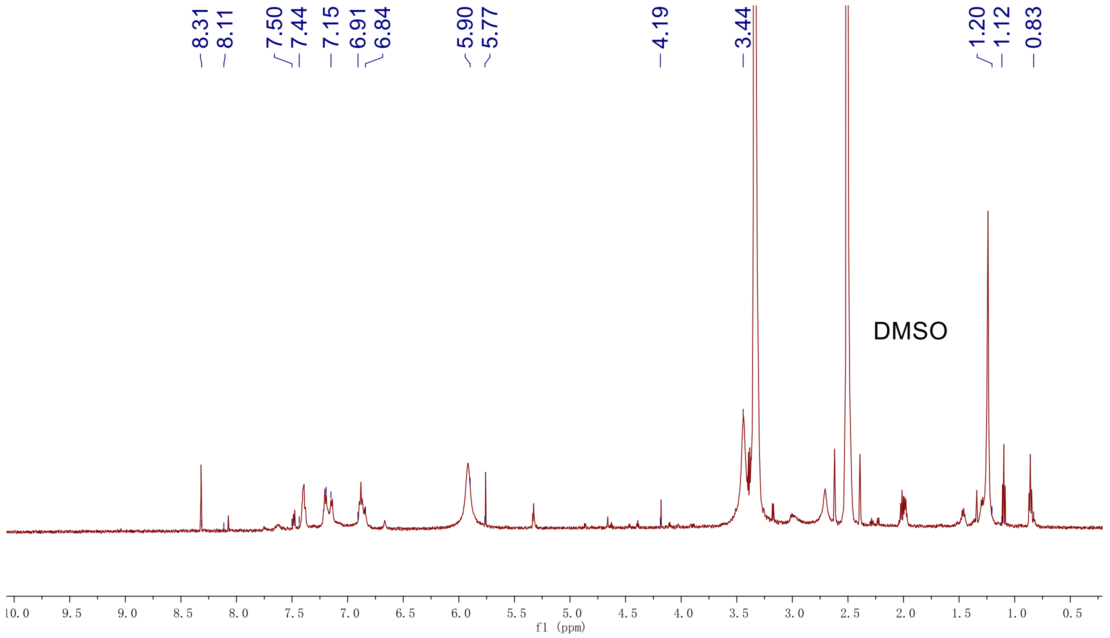

Figure S14. ^1H NMR of $Ag_{17}Cu_{10}H_4$ cluster in d_6-DMSO, related to the Figure 3.

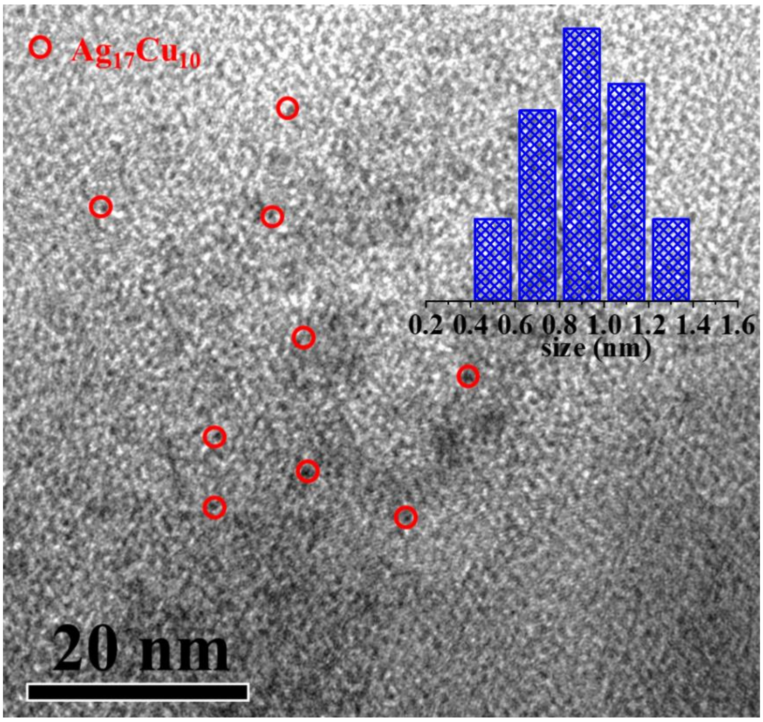

Figure S15. TEM images of XC-72R-supported $Ag_{17}Cu_{10}H_4$ clusters (1 wt%), related to the Figure 5.

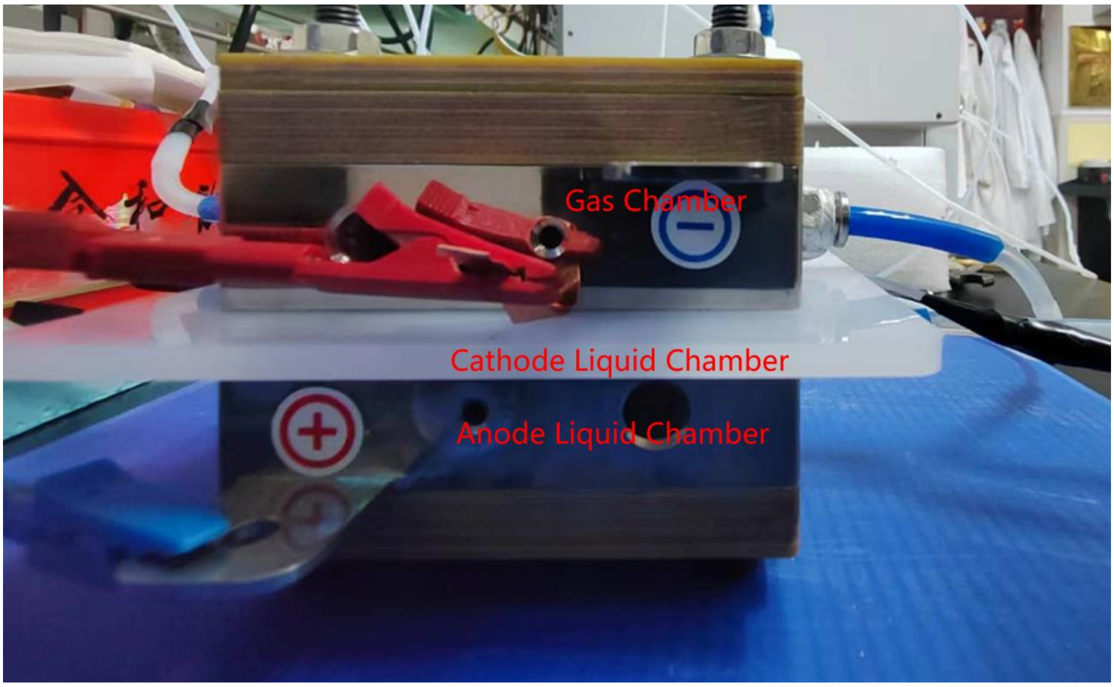

Figure S16. Equipment for electrochemical CO_2 reduction in this work, related to the Figure 5.

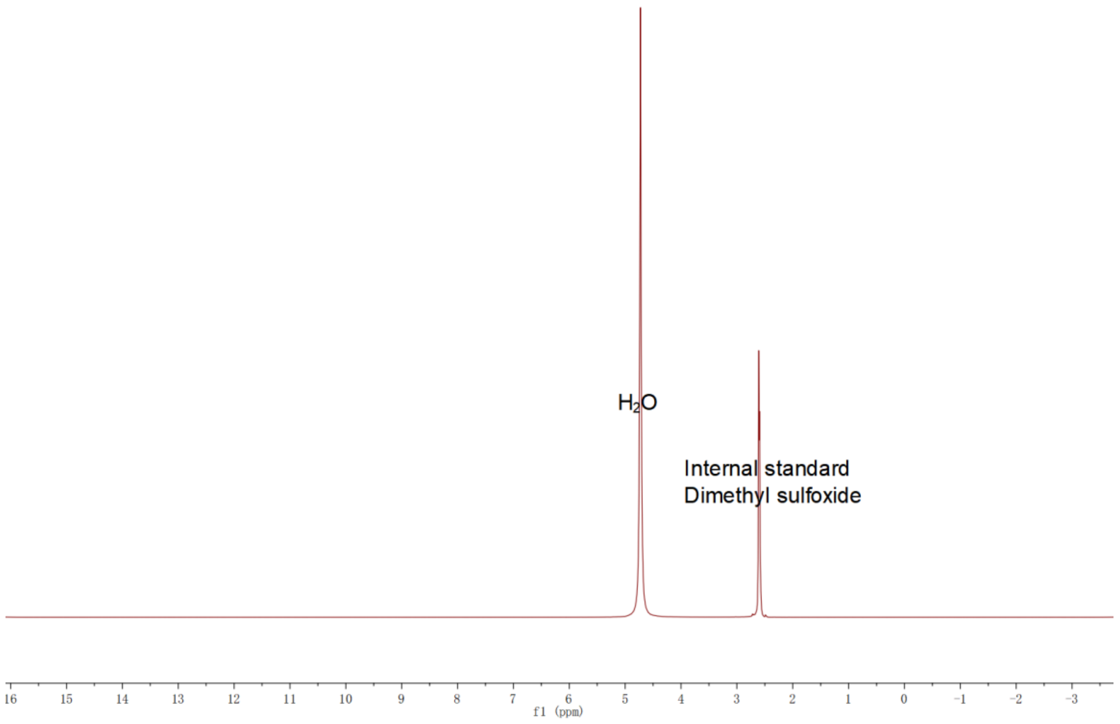

Figure S17. ^{1}H NMR of liquid product after the CO_2 electroreduction by $Ag_{17}Cu_{10}H_4$, related to the Figure 5. No characteristic peak of HCOOH was observed.

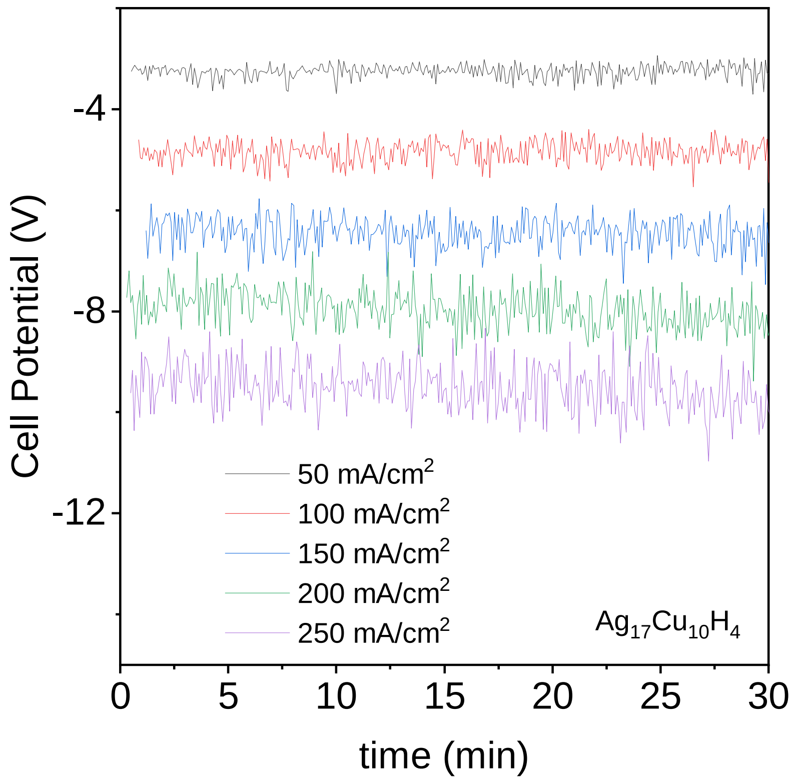

Figure S18. Chronopotentiometry tests of $Ag_{17}Cu_{10}H_4$, related to the Figure 5.

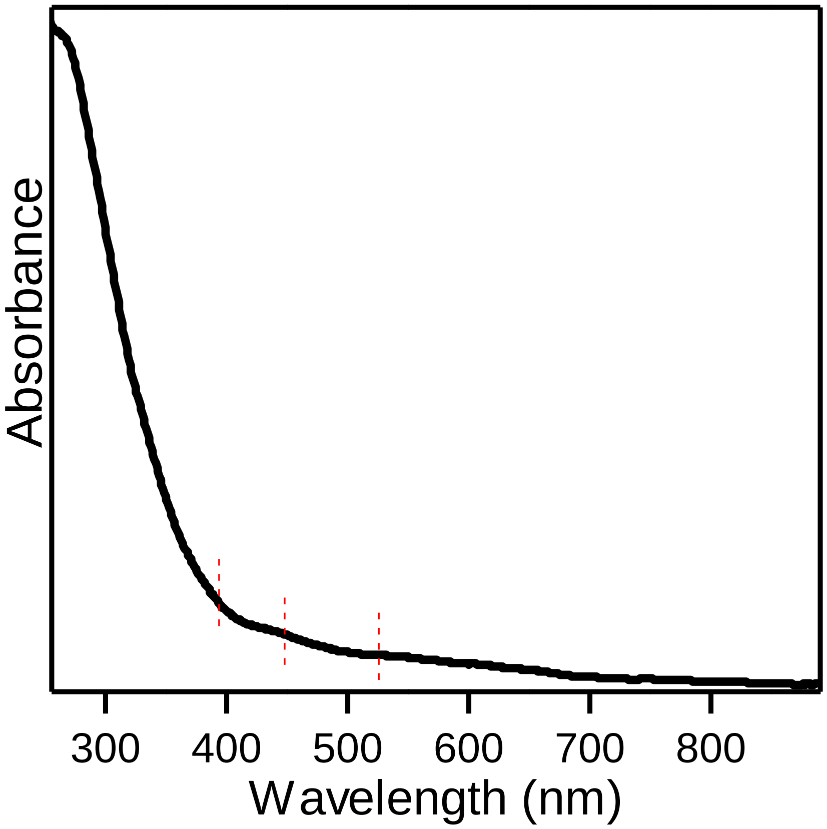

Figure S19. The UV-Vis spectrum of $Ag_{17}Cu_{10}H_4$ after electrocatalytic CO_2 reduction, related to the Figure 5. The clusters were recovered by immersing the carbon paper in dichloromethane and sonicating it for 5 minutes. The presence of the peaks at 390, 446 and 524 nm indicate the intact $Ag_{17}Cu_{10}H_4$ nanoclusters.

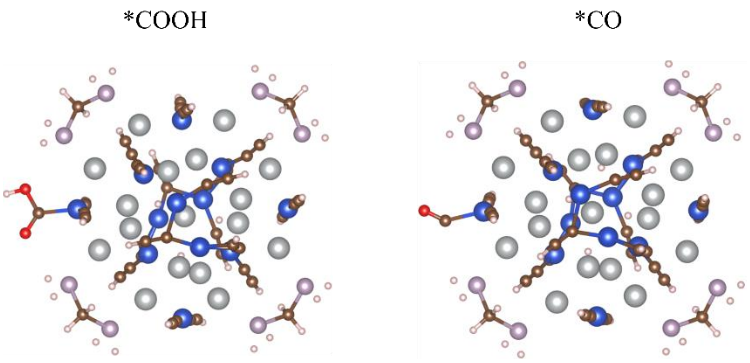

Figure S20. DFT images showing that reaction intermediates of both *COOH and *CO can exist stably at the Cu site, related to the Figure 6.

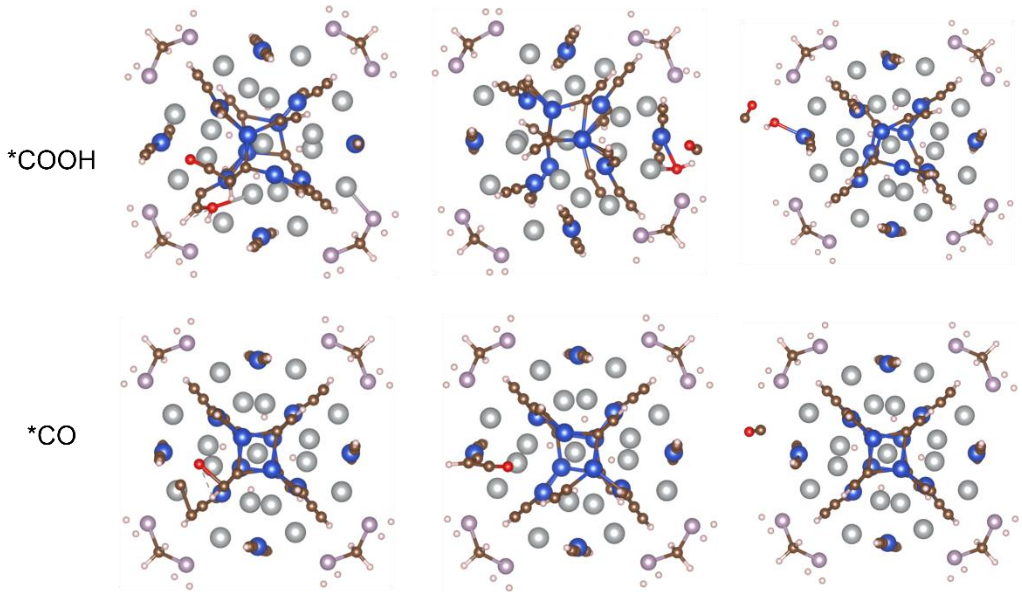

Figure S21. DFT images showing that reaction intermediates of *COOH and *CO cannot exist stably at the Ag site, related to the Figure 6.

Table S1. Crystallographic data of $Ag_{17}Cu_{10}H_4$, related to the Figure 1.

identification code	$[Ag_{17}Cu_{10}(dppm)_4(PA)_{20}H_4]^{3+}$
formula	$C_{276}H_{224}Ag_{17}Cl_{32}Cu_{10}P_8$
formula weight	7391.89
Temperature/K	100(10)
crystal system	cubic
space group	$I\text{-}\bar{4}3m$
a (Å)	37.74904(17)
b (Å)	37.74904(17)
c (Å)	37.74904(17)
α (˚)	90
β (˚)	90
γ (˚)	90
V (Å^3)	53792.0(7)
Z	6
D_c / (g·cm^{-3})	1.369
Radiation	Cu Kα (λ= 1.54184 Å)
Theta (˚) range	4.682 to 134.692
Index ranges	-33 ≤ h ≤ 44, -45≤ k ≤ 44, -45 ≤ l ≤ 45
Refls. Total	310622
restraints	602
parameters	362
R_{int}	0.0811
R_1/wR_2 [I>2σ(I)]	0.0727/0.2290
R_1/wR_2 (all data)	0.0941/0.2569
GooF	1.072

Table S2. Selected bond lengths (Å) for compound $Ag_{17}Cu_{10}H_4$, related to the Figure 2.

Parameter	value	Parameter	value
Ag2-Ag2	2.792(3)	Ag3-C9	2.431(16)
Ag2-Ag4	3.2005(18)	Ag3-C17	2.502(19)
Ag2-Cu1	2.845(3)	Ag4-Cu1	2.740(3)
Ag2-Cu1	2.849(3)	Ag4-Cu3	3.093(5)
Ag2-H	1.9365(18)	Ag4-Cu1	2.740(3)
Ag2-Cu2	2.804(3)	Cu1-H	2.073(7)
Ag2-Cu3	2.599(4)	Cu1-C17	2.03(2)
Ag2-C1	2.291(15)	Cu1-C25	1.78(2)
Ag2-C9	2.298(16)	Cu2-H	2.07(2)
Ag2-C25	2.70(2)	Cu2-C9	1.824(14)
Ag3-Ag3	2.976(2)	Cu3-Cu3	1.361(12)
Ag3-Cu1	2.793(3)	Cu3-C1	1.889(17)
Ag3-Cu2	2.8720(11)	Cu3-C17	2.33(2)
Ag3-P1	2.387(4)		

Table S3. Optimized Fractional Atomic Coordinates for $Ag_{17}Cu_{10}H_4$, related to the Figure 4.

Atom		x	y	z	Atom		x	y	z
1	Ag	3.625279	0.389975	3.494702	87	H	-3.61255	-3.55295	-4.75301
2	Ag	7.580639	-2.423	1.126197	88	H	3.884948	2.515128	-5.77261
3	Ag	-4.0328	-2.05714	2.331628	89	H	3.899836	-4.56735	5.387884
4	Ag	-7.95268	2.500294	1.531036	90	C	3.631127	-2.73122	-0.68469
5	Ag	-4.45301	0.814735	-2.61373	91	C	4.874977	-2.22798	-1.36652
6	Ag	-5.98126	-4.16655	-1.53106	92	C	-2.70139	3.15824	0.45951
7	Ag	2.392915	0.353512	-3.06582	93	C	-3.42252	4.048256	-0.49364
8	Ag	7.215117	4.756157	-0.31369	94	C	-2.95767	-3.67634	1.544953
9	Ag	0.491217	-3.76555	3.356976	95	C	-3.32294	-4.86582	2.377844
10	Ag	5.216905	-5.97075	0.396125	96	C	3.163087	3.949919	1.891935
11	Ag	-0.87356	3.093855	2.589113	97	C	2.458766	4.271515	3.163305
12	Ag	-6.18471	6.358334	0.89199	98	H	9.504544	-3.69278	2.290322
13	Ag	-0.06441	2.962992	-2.36271	99	H	-10.3242	3.201714	0.948133
14	Ag	4.191878	7.174112	-2.01135	100	H	-7.40041	-5.95069	-2.74344
15	Ag	1.082266	-3.70099	-3.2378	101	H	8.269011	5.823124	-2.38248
16	Ag	-2.62898	-6.60163	-1.02428	102	H	7.061822	-8.76714	-1.71281
17	Ag	0.084801	0.066948	1.129995	103	H	6.443162	-8.33635	-1.86808
18	Cu	2.883578	-1.69994	0.957303	104	H	-7.8619	7.493194	2.42246
19	Cu	-3.20046	1.08489	0.650337	105	H	1.543141	8.537195	-0.01386
20	Cu	-0.86366	-2.44436	-0.70402	106	H	1.596743	7.825219	-0.29452
21	Cu	3.100587	2.727284	-0.62813	107	H	6.063259	8.095277	-3.47625
22	H	1.138402	0.03022	-1.14992	108	H	-3.73588	-7.83287	-2.98197
23	Cu	4.399857	0.751467	-0.40668	109	C	8.011427	-5.48017	1.107312
24	H	-0.72845	1.192233	-1.05808	110	H	8.579647	-6.14617	1.843767
25	Cu	-2.52703	-0.88566	-0.45447	111	H	8.739846	-5.57541	0.250145
26	H	0.774522	-0.47908	-1.60552	112	C	-8.91891	5.334824	1.364079
27	Cu	1.20014	-3.1018	0.150065	113	H	-9.75992	5.839649	1.922605
28	H	-0.65707	1.794295	-0.56393	114	H	-9.1557	5.512356	0.297394
29	Cu	1.68559	4.166635	0.460293	115	C	-5.61288	-7.12677	-1.29878
30	Cu	0.888413	-1.02437	5.450826	116	H	-6.16202	-7.95951	-1.84908
31	Cu	-3.72459	1.299338	2.924896	117	H	-5.9363	-7.39539	-0.25439
32	Cu	-1.90359	-1.62054	-2.59806	118	C	7.001886	7.694816	-1.03775
33	Cu	-0.40146	0.664634	-4.84869	119	H	7.794616	8.421534	-1.39803
34	P	8.074599	-3.68073	1.920196	120	H	6.814509	8.069561	-0.00244
35	P	-9.15567	3.431001	1.826231	121	H	-4.50392	4.060157	-0.28399
36	P	-6.36199	-5.39705	-1.8267	122	H	3.202643	4.275803	3.99954
37	P	7.928644	5.971094	-0.95107	123	H	5.416982	-3.07449	-1.84327
38	P	6.437257	-6.45894	0.62451	124	H	-3.30633	-4.65649	3.472782
39	P	-7.27385	6.225184	1.94605	125	H	-0.06638	2.696837	5.526614
40	P	5.43462	8.097809	-2.14208	126	H	1.512651	1.98547	5.098345

41	P	-3.71231	-7.54765	-1.5327	127	H	-2.01055	-2.09532	4.731236
42	C	-0.1301	0.585804	5.174785	128	H	-2.04434	-1.05786	3.279111
43	C	0.565174	1.799096	5.62951	129	H	-3.04705	2.854179	-6.35568
44	H	0.803166	1.762108	6.706968	130	H	-1.3345	3.206324	-6.1285
45	C	-0.94903	-0.25411	4.862784	131	H	1.207899	-1.20803	-6.43671
46	C	-2.04831	-1.03779	4.381606	132	H	-0.17976	-1.02028	-7.43059
47	H	-2.99756	-0.66793	4.809744	133	H	6.888483	0.141641	5.816626
48	C	-2.13857	1.341439	-5.26733	134	H	7.434435	-1.16471	6.76675
49	C	-2.18691	2.706674	-5.63724	135	H	-7.88968	-2.08103	1.606023
50	H	-2.50797	3.436181	-4.85263	136	H	-8.62613	-0.57171	1.154158
51	C	-0.72083	-1.14417	-5.33153	137	H	-8.33106	-2.37629	-2.98446
52	C	0.113136	-1.47029	-6.45276	138	H	-6.69092	-1.88059	-3.50509
53	H	0.120047	-2.5604	-6.6824	139	H	7.243375	1.529941	-2.61016
54	C	6.72167	-1.7684	4.848785	140	H	5.935682	2.17438	-3.52808
55	C	6.675067	-0.96907	5.946111	141	H	1.20978	-7.08471	-0.64851
56	H	5.740806	-0.96336	6.597476	142	H	-0.10071	-6.25325	0.251673
57	C	-7.24869	-1.26789	-0.25376	143	H	-2.89977	7.828381	3.012147
58	C	-7.64549	-1.10436	1.120069	144	H	-1.58866	6.861434	3.369936
59	H	-6.94789	-0.54691	1.760465	145	H	-0.14063	5.663349	-5.23075
60	C	-7.15939	-1.59719	-1.39532	146	H	0.690188	7.124948	-5.32028
61	C	-7.50803	-1.65892	-2.81003	147	H	0.983526	-7.37383	-4.87586
62	H	-7.97996	-0.69191	-3.11898	148	H	-0.65286	-7.13108	-4.23607
63	C	5.554671	1.968573	-1.44066	149	H	3.414984	-2.92969	5.477947
64	C	6.123431	1.473499	-2.65129	150	H	5.002107	-3.25729	5.737579
65	H	5.948503	0.472856	-3.07736	151	H	-3.9611	3.742093	4.684993
66	C	1.513908	-4.99726	-0.44148	152	H	-5.62819	4.068664	4.304574
67	C	0.98401	-6.22586	0.035624	153	H	-2.7323	-4.95723	-4.14016
68	H	1.404844	-6.58935	1.009099	154	H	-1.86529	-3.5575	-4.76964
69	C	-2.28595	6.652213	1.344176	155	H	3.417547	3.914075	-6.47708
70	C	-2.49791	6.823421	2.686683	156	H	2.165874	2.997883	-5.87256
71	H	-3.22048	6.14122	3.239389	157	H	3.82242	-3.74892	-0.29017
72	C	1.924093	5.516866	-4.69483	158	H	2.877486	-2.78701	-1.49214
73	C	0.747233	6.168222	-4.71258	159	H	5.588446	-1.7067	-0.71795
74	H	0.307464	6.623385	-3.76007	160	H	4.636623	-1.57219	-2.22871
75	C	0.83694	-7.70402	-2.76867	161	H	-2.86938	3.535858	1.491897
76	C	0.300499	-7.6974	-4.02348	162	H	-1.63748	3.026725	0.181748
77	H	0.001659	-8.72061	-4.42206	163	H	-3.29512	3.759686	-1.54778
78	C	4.544354	-3.58356	3.667961	164	H	-3.04732	5.091976	-0.43212
79	C	4.225732	-3.52992	4.982429	165	H	-1.94143	-3.31604	1.742178
80	C	-4.79962	2.907342	2.782628	166	H	-3.19481	-3.759	0.484922
81	C	-4.61829	3.898565	3.811927	167	H	-2.59504	-5.68913	2.253931
82	C	-2.85213	-3.29744	-2.80132	168	H	-4.32164	-5.27104	2.159349
83	C	-2.72607	-3.84163	-4.13526	169	H	3.553597	2.918528	1.893716

84	C	3.336538	4.043096	-4.36138	170	H	3.922547	4.691321	1.59174
85	C	3.16012	3.377743	-5.52904	171	H	1.703899	3.540383	3.484035
86	H	-4.40135	4.919504	3.41451	172	H	2.024002	5.27652	3.211604

Table S4. **Optimized Bond Lengths for $Ag_{17}Cu_{10}H_4$ cluster, related to the Figure 4**.

Tag	Atoms	Length/Å	Tag	Atoms	Length/Å
1-2	Ag-Ag	5.400697	19-92	Cu-C	2.14109
4-5	Ag-Ag	5.680481	92-93	C-C	1.490184
3-6	Ag-Ag	4.813155	3-94	Ag-C	2.096797
3-9	Ag-Ag	4.943355	94-95	C-C	1.49733
2-10	Ag-Ag	4.32513	29-96	Cu-C	2.06871
1-11	Ag-Ag	5.326403	96-97	C-C	1.488581
4-12	Ag-Ag	4.291685	34-98	P-H	1.477119
7-13	Ag-Ag	3.652698	35-99	P-H	1.479537
8-14	Ag-Ag	4.22712	36-100	P-H	1.491728
7-15	Ag-Ag	4.264548	37-101	P-H	1.47874
6-16	Ag-Ag	4.174227	39-104	P-H	1.476693
11-17	Ag-Ag	3.49423	40-107	P-H	1.474852
17-18	Ag-Cu	3.31434	41-108	P-H	1.477256
17-19	Ag-Cu	3.47264	38-109	P-C	1.915491
17-20	Ag-Cu	3.251129	109-110	C-H	1.144028
7-21	Ag-Cu	3.475333	109-111	C-H	1.128893
7-22	Ag-H	2.31279	39-112	P-C	1.958997
21-23	Cu-Cu	2.375079	112-113	C-H	1.128756
13-24	Ag-H	2.297518	112-114	C-H	1.106979
20-25	Cu-Cu	2.293173	36-115	P-C	1.9575
7-26	Ag-H	2.333425	115-116	C-H	1.139227
20-27	Cu-Cu	2.328292	115-117	C-H	1.125833
13-28	Ag-H	2.225473	37-118	P-C	1.958982
21-29	Cu-Cu	2.293166	118-119	C-H	1.134169
19-31	Cu-Cu	2.343997	118-120	C-H	1.116872
20-32	Cu-Cu	2.312475	93-121	C-H	1.101601
7-33	Ag-Cu	3.329256	97-122	C-H	1.119224
2-34	Ag-P	1.567262	91-123	C-H	1.112489
4-35	Ag-P	1.549372	95-124	C-H	1.114892
6-36	Ag-P	1.321544	43-125	C-H	1.102444
8-37	Ag-P	1.546431	43-126	C-H	1.102082
10-38	Ag-P	1.334074	46-127	C-H	1.114469
12-39	Ag-P	1.521511	46-128	C-H	1.102684
14-40	Ag-P	1.553936	49-129	C-H	1.130369
16-41	Ag-P	1.525467	49-130	C-H	1.10345
30-42	Cu-C	1.92516	52-131	C-H	1.12585
42-43	C-C	1.470461	52-132	C-H	1.115549
43-44	C-H	1.104049	55-133	C-H	1.138417
42-45	C-C	1.213855	55-134	C-H	1.135061
45-46	C-C	1.433214	58-135	C-H	1.117889
46-47	C-H	1.105068	58-136	C-H	1.116476

33-48	Cu-C	1.910722	61-137	C-H	1.105628
48-49	C-C	1.415288	61-138	C-H	1.095404
49-50	C-H	1.118425	64-139	C-H	1.122119
33-51	Cu-C	1.899183	64-140	C-H	1.138082
51-52	C-C	1.434926	67-141	C-H	1.120992
52-53	C-H	1.114066	67-142	C-H	1.106363
54-55	C-C	1.35839	70-143	C-H	1.130208
55-56	C-H	1.138927	70-144	C-H	1.13798
57-58	C-C	1.439308	73-145	C-H	1.145291
58-59	C-H	1.098868	73-146	C-H	1.134844
57-60	C-C	1.191459	76-147	C-H	1.139196
60-61	C-C	1.458344	76-148	C-H	1.129067
61-62	C-H	1.119499	79-149	C-H	1.123891
23-63	Cu-C	1.970799	79-150	C-H	1.116842
63-64	C-C	1.426265	81-151	C-H	1.103916
64-65	C-H	1.101553	81-152	C-H	1.136451
27-66	Cu-C	2.010264	83-153	C-H	1.115634
66-67	C-C	1.42052	83-154	C-H	1.106392
67-68	C-H	1.121106	85-155	C-H	1.11924
69-70	C-C	1.369876	85-156	C-H	1.118403
70-71	C-H	1.137101	90-157	C-H	1.108134
72-73	C-C	1.345206	90-158	C-H	1.105922
73-74	C-H	1.143613	91-159	C-H	1.09609
75-76	C-C	1.364686	91-160	C-H	1.109162
76-77	C-H	1.138037	92-161	C-H	1.112043
78-79	C-C	1.353596	92-162	C-H	1.107405
31-80	Cu-C	1.939485	93-163	C-H	1.100329
80-81	C-C	1.440437	93-164	C-H	1.110816
32-82	Cu-C	1.937267	94-165	C-H	1.096114
82-83	C-C	1.446188	94-166	C-H	1.089374
84-85	C-C	1.355453	95-167	C-H	1.105912
81-86	C-H	1.116835	95-168	C-H	1.099704
83-87	C-H	1.118392	96-169	C-H	1.102846
85-88	C-H	1.152739	96-170	C-H	1.102984
79-89	C-H	1.160549	97-171	C-H	1.098748
18-90	Cu-C	2.078103	97-172	C-H	1.096079
90-91	C-C	1.505093			

Table S5. Comparison of Optimized and Experimental Bond Lengths for $Ag_{17}Cu_{10}H_4$, related to the Figure 4.

Atom	Atom	Optimized average length/ Å	Atom	Atom	Experimental average length/ Å
Ag	Ag	4.549	Ag	Ag	2.9895
Ag	Cu	3.369	Ag	Cu	2.8639
Cu	Cu	2.324	Cu	Cu	1.361
Ag	C	2.097	Ag	C	2.3805
Ag	H	2.292	Ag	H	1.9365
Ag	P	1.490	Ag	P	2.3876
Cu	C	1.988	Cu	C	1.938
C	P	1.947	C	P	1.877
C	C	1.405	C	C	1.3889
C	H	1.118	C	H	0.9500

Table S6. Frontier orbitals for $Ag_{17}Cu_{10}H_4$, related to the Figure 4. Gap=2.15 eV.

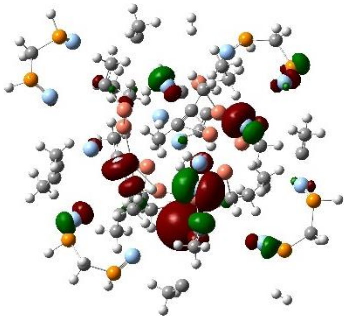<br>HOMO-5<br>-10.69 eV	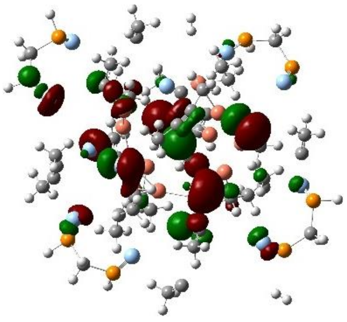<br>HOMO-4<br>-10.51 eV	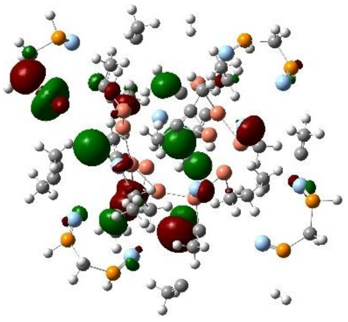<br>HOMO-3<br>-10.44 eV
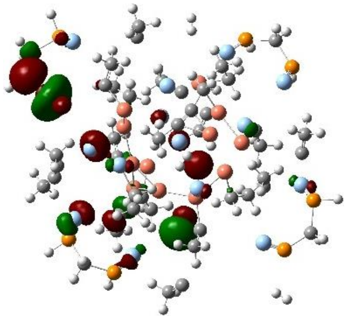<br>HOMO-2<br>-10.40 eV	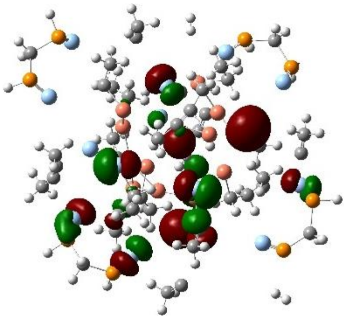<br>HOMO-1<br>-10.16 eV	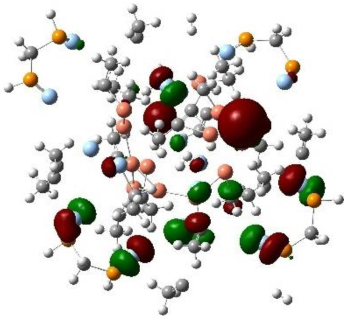<br>HOMO<br>-9.97 eV
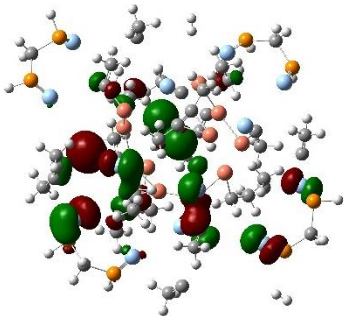<br>LUMO<br>-7.82 eV	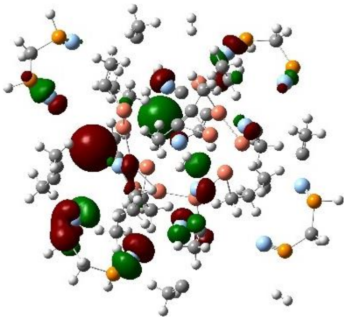<br>LUMO+1<br>-7.56 eV	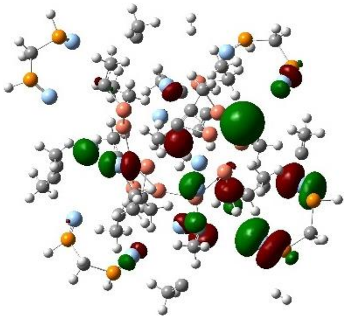<br>LUMO+2<br>-7.46 eV
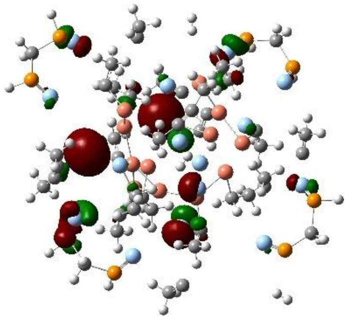<br>LUMO+3<br>-7.31 eV	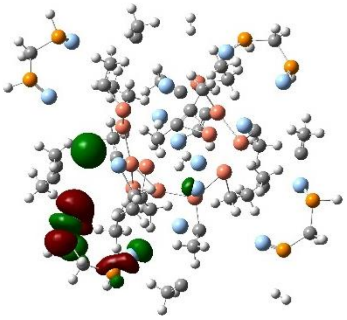<br>LUMO+4<br>-7.09 eV	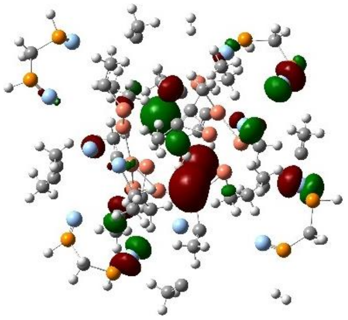<br>LUMO+5<br>-6.97 eV

Table S7. Bader charge of each atomic group of $Ag_{17}Cu_{10}H_4$, related to the Figure 6.

Atomic group	Bader
Ag	0.309
Cu	0.438
P	1.286
C	-0.402
H	-0.065
